# Exposure of neonatal rats to alcohol has differential effects on neuroinflammation and neuronal survival in the cerebellum and hippocampus

**DOI:** 10.1186/s12974-015-0382-9

**Published:** 2015-09-04

**Authors:** Lauren A. Topper, Brian C. Baculis, C. Fernando Valenzuela

**Affiliations:** Department of Neurosciences, School of Medicine, MSC08 4740, University of New Mexico Health Sciences Center, Albuquerque, NM 87131-0001 USA

**Keywords:** Microglia, Cytokines, Astrocytes, Alcohol, Neurodegeneration, Fetal, Cerebellum, Hippocampus, Development

## Abstract

**Background:**

Fetal alcohol exposure is a leading cause of preventable birth defects, yet drinking during pregnancy remains prevalent worldwide. Studies suggest that activation of the neuroimmune system plays a role in the effects of alcohol exposure during the rodent equivalent to the third trimester of human pregnancy (i.e., first week of neonatal life), particularly by contributing to neuronal loss. Here, we performed a comprehensive study investigating differences in the neuroimmune response in the cerebellum and hippocampus, which are important targets of third trimester-equivalent alcohol exposure.

**Methods:**

To model heavy, binge-like alcohol exposure during this period, we exposed rats to alcohol vapor inhalation during postnatal days (P)3–5 (blood alcohol concentration = 0.5 g/dL). The cerebellar vermis and hippocampus of rat pups were analyzed for signs of glial cell activation and neuronal loss by immunohistochemistry at different developmental stages. Cytokine production was measured by reverse transcriptase polymerase chain reaction during peak blood alcohol concentration and withdrawal periods. Additionally, adolescent offspring were assessed for alterations in gait and spatial memory.

**Results:**

We found that this paradigm causes Purkinje cell degeneration in the cerebellar vermis at P6 and P45; however, no signs of neuronal loss were found in the hippocampus. Significant increases in pro-inflammatory cytokines were observed in both brain regions during alcohol withdrawal periods. Although astrocyte activation occurred in both the hippocampus and cerebellar vermis, microglial activation was observed primarily in the latter.

**Conclusions:**

These findings suggest that heavy, binge-like third trimester-equivalent alcohol exposure has time- and brain region-dependent effects on cytokine levels, morphological activation of microglia and astrocytes, and neuronal survival.

**Electronic supplementary material:**

The online version of this article (doi:10.1186/s12974-015-0382-9) contains supplementary material, which is available to authorized users.

## Background

Drinking during pregnancy can result in fetal alcohol spectrum disorders (FASDs), an umbrella term used to describe a wide array of teratogenic effects that occur in an estimated 2–5 % of live births in the USA [1]. FASDs range from the most severe form, fetal alcohol syndrome, to less obvious neurocognitive deficits and behavioral abnormalities. Although the mechanisms underlying FASD are not fully understood, one of the most detrimental effects of alcohol exposure in the developing brain is neuronal loss [2–4]. Recently, activation of the neuroimmune system has been observed in parallel with neurodegeneration, and this effect has been suggested to play a central role in the pathophysiology of FASD.

The neuroimmune system is comprised primarily of microglia and astrocytes, although microglia are much better characterized with respect to this system. Along with their critical functions in defending the CNS from damage, astrocytes and microglia have important roles during development, particularly in synaptic refinement [5, 6]. Both cell types undergo maturation concurrent with the brain growth spurt (reviewed by [7, 8]), which occurs during the third trimester of human pregnancy and is equivalent to the early postnatal period in rodents, suggesting that these cells may be particularly vulnerable to insult during this time. While considerable research has been conducted into alcohol’s neuroinflammatory effects in adolescents and adults [9–18], the unique neuroimmune response to alcohol during development has only begun to be investigated.

Research focused on prenatal alcohol exposure (i.e., first and second trimester-equivalent exposure) has indicated an increased number of microglia/macrophage throughout brain white matter [19] and in the cerebellum [20] and a reduced number of Bergmann glial cells [21]. Models utilizing cell culture or in vivo prenatal exposure have shown that astrocytes can contribute to repair processes engaged in response to alcohol exposure, including promoting neuronal survival, dendritic outgrowth, and plasticity (reviewed by [22]).

Additionally, studies in models of third trimester-equivalent alcohol exposure have demonstrated elevations in pro-inflammatory cytokines in multiple brain regions [23, 24], which were long-lasting in some cases [25] and widespread signs of neuronal loss [4]. Moreover, blunting neuroinflammation correlated with decreased neuronal loss [26, 24] and improved performance in hippocampal-dependent tasks [25]. Based on these results, it was concluded that neuroimmune activation contributes to neuronal loss across several brain regions. Additionally, while postnatal alcohol exposure (PAE) has been shown to activate microglia [26, 27], a potential role for astrocytes in the neuroimmune response to PAE has received little attention, despite the fact that astrocytes commonly secrete cytokines in response to CNS insults (reviewed by [28]) and regulate microglial activation (Reviewed by [29]). Furthermore, a more comprehensive analysis of the neuroimmune response to multiple alcohol exposures during development, including the individual effect of withdrawal periods, has yet to be undertaken.

In this study, we further characterized the effect of alcohol exposure during the third trimester-equivalent on the neuroimmune system. To investigate the relationship between neuronal loss and neuroimmune activation, we utilized a paradigm designed to induce neurodegeneration and compared the respective neuroimmune responses in the cerebellum and hippocampus. We included end points collected during periods of both peak blood alcohol levels and withdrawal. These end points incorporate an investigation into both astrocyte and microglial activations with an in-depth analysis of the specific layers in which activation of these cells occurs, with respect to neuronal loss. In addition, we measured mRNA levels for both pro- and anti-inflammatory cytokines.

## Materials and methods

### Animal treatments

Animal procedures were approved by the Institutional Animal Care and Use Committee of the University of New Mexico Health Sciences Center. Time-pregnant Sprague-Dawley rats were obtained from Harlan Laboratories Inc. (Indianapolis, IN) and allowed to acclimate for 1 week before giving birth. Only male offspring were used for experiments, as developmental alcohol exposure has been shown to have sexually dimorphic effects [30–32] and microglial colonization of the brain is different in males and females [33]. Previous studies have demonstrated that binge-like exposure to high levels of alcohol is effective at inducing neuronal cell loss in the rodent brain [4]. Additionally, binge-like, heavy alcohol-exposure paradigms in the early postnatal period have been shown to be more effective in generating a robust neuroimmune response [24–26] when compared to more moderate but long-term exposures [34]. Importantly, heavy alcohol exposure during late pregnancy has been documented in humans [35, 36]. We chose to expose pups to higher levels of alcohol than those typically achieved in humans because the developing rodent brain is comparatively more resistant to alcohol than the human brain [37, 38]. We used a vapor inhalation paradigm because it is less invasive than other methods, requires minimum pup handling, and allows pups to remain with their mothers throughout exposure.

**Fig. 1:**
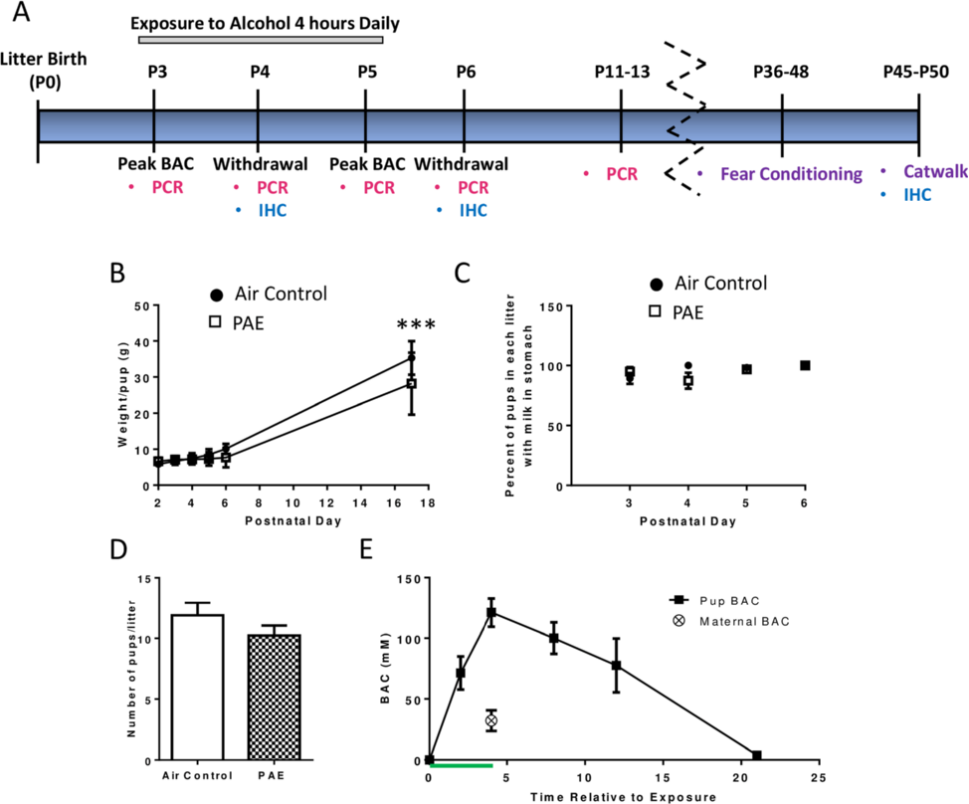
Characterization of the exposure paradigm. **a** Time line for alcohol exposures and experiments. Pups, along with dams, were exposed to alcohol for 4 h/day via vapor inhalation from P3 to P5 (*gray bar*). Samples collected from P3–P6 were taken either during periods of withdrawal or peak blood alcohol concentrations (BAC) as indicated and were used for PCR (*pink text*) and/or immunohistochemistry (IHC, *blue text*). Each collected IHC time point included staining for IBA-1/microglia, GFAP/astrocytes, and neuronal loss. IHC was also performed for neuronal apoptosis but only on P4 and only in the cerebellar vermis region. Additional time points were included beyond the exposure paradigm for PCR, IHC, and behavior (*purple text*). **b** Pups were weighed daily and the average weight per pup was recorded (*n* = 3–12 litters). **c** Nursing ability was quantified as the percentage of pups in each litter with milk visible in their stomachs each morning prior to exposure (*n* = 5–6 litters). **d** Similar litter sizes were assigned to each treatment group on P3 (*n* = 17 litters). **e** Pup blood alcohol concentrations (*n* = 4 pups from 4 litters) were each measured at the beginning (0 h), middle (2 h), and end (4 h) of the exposure, as well as at several time points after. The *green line* represents the 4-h exposure period. Peak maternal BAC was assessed at 4 h (*n* = 3 dams exposed after pups had been weaned). (***p < 0.001)

Three days after litter birth, dams and their respective offspring were housed together in the vapor chamber apparatus. Litters were randomly assigned to either alcohol treatment or control. Starting at 10:00 a.m., litters were exposed to alcohol via vapor inhalation for 4 h daily during their light cycle, from postnatal day (P)3 through P5, a period of exposure that has previously been shown to activate the neuroimmune system [26] (Fig. 1a). Alcohol vapor chamber levels were 8.03 g/dL ± 0.21 at 4 h (*n* = 9 rounds of exposure). Control litters were housed in identical chambers that had only air flowing through them. Pups were handled daily only for weighing purposes. Blood alcohol concentrations (BACs) were determined using an alcohol dehydrogenase-based assay, as previously described [39]. On P4, BACs were measured at 0, 2, 4, 8, 12, and 21 h after exposure began, with 0 h being the time immediately preceding exposure. Maternal BACs were also measured in a separate group of dams subjected to the 4-h exposure paradigm after pups had been weaned.

Nursing ability was assessed by counting the number of pups in each litter who had a visible presence of milk in their stomachs on the mornings after each exposure day. Finally, maternal care was assessed as previously described [40]. Briefly, on P4, starting at exposure hour 0, litters were filmed for 15 min out of every hour during the 4-h exposure, plus 4 additional hours following exposure (8 total hours). Maternal behavior was scored by a blinded observer every 3 min during each 15 min clip for the following actions: no contact with the pups, arched-back nursing, blanket nursing, passive nursing, licking pups, and licking pups during arched-back nursing.

### Reverse transcriptase polymerase chain reaction

Samples were collected during peak BACs on P3 and P5 (within 30 min following the 4-h alcohol exposure) and during periods of alcohol withdrawal on P4 and P6 (Fig. 1a). Additionally, to address the duration of effects, samples were collected at P11–13. Eight pups (from a total of 8 litters) were used per treatment group. Animals were anesthetized with ketamine (250 mg/kg). Whole hippocampi and cerebellar vermises were collected. Tissue was homogenized by sonication in ice cold RNeasy Lysis Buffer and RNA was extracted using an RNeasy Mini Kit according to the manufacturer’s instructions (SABiosciences/Qiagen, Valencia, CA). The RNA was stored at −20 °C until use. cDNA was synthesized from 1 μg of RNA using a High Capacity cDNA Reverse Transcription Kit (Applied Biosystems, cat# 4368813, Foster City, CA) according to the manufacturer’s instructions. The following primers from SABiosciences/Qiagen were used: interleukin-1β (IL-1β, cat# PPR06480B), tumor necrosis factor α (TNFα, cat# PPR06411F), interleukin-10 (IL10, cat# PPR06479A), transforming growth factor β (TGFβ, cat# PPR06430B), and hypoxanthine phosphoribosyltransferase 1(HPRT1, cat# PPR42247F). Only samples with threshold cycle values (CT) under 35 were used for analysis. All values were normalized to HPRT1 via the ΔΔCT method. HPRT1 was not affected by PAE at any age in either the cerebellar vermis (interaction F(_4,70_) = 0.22, *p* = 0.929; alcohol F(_1,70_) = 0.20; *p* = 0.657; age F(_4,70_) = 2.50, *p* = 0.050, *n* = 8 pups from 8 litters) or the hippocampus (interaction F(_4,69_) = 0.36, *p* = 0.836; alcohol F(_1,69_) = 0.003, *p* = 0.956; age F(_4,69_) = 13.76, *p* < 0.0001, *n* = 7–8 pups from 7–8 litters). Then, for each cytokine, the average value of the P3 air control was computed and each individual data set was divided by this average, including the P3 air control that was used to compute the average.

### Immunohistochemistry

On P4, P6, and P45 (Fig. 1a), 4 pups (from a total of 4 litters) per treatment group were anesthetized by injection of ketamine (250 mg/kg). Animals were transcardially perfused first with phosphate buffered saline (PBS), pH 7.4, containing procaine hydrochloride (1 g/L) and heparin (1 USP unit/L) for 4 min, followed by ice cold 4 % paraformaldehyde in PBS. Brains were removed by decapitation and placed in 4 % paraformaldehyde for 48 h at 4 °C, then 30 % sucrose for 24–48 h at 4 °C. Brains were embedded in Optimal Cutting Temperature compound (Fisher Healthcare, Houston, TX) and frozen before sectioning on a cryostat (Microm, model# HM 505E, Walldorf, Germany) at 16 μm. Sections for staining were incubated with PBS containing 1 % bovine serum albumin, 0.2 % Triton X-100, and 5 % of either donkey or goat serum to match the host species of the secondary antibodies described below. Primary antibodies were applied overnight at 4 °C. Astrocytes were stained with rabbit anti-glial fibrillary acidic protein (GFAP, 1:500, Dako, cat# 019-19741, Carpinteria, CA), microglia with rabbit anti-ionized calcium-binding adapter molecule 1 (IBA-1, 1:500, Wako, ref#Z0334, Richmond, VA), Purkinje cells with mouse anti-Calbindin (1:500, Santa Cruz, cat# sc-70478, Dallas, TX), hippocampal neurons with mouse anti-NeuN (1:500, Millipore, cat# MAB377A5, Bedford, MA), and cell nuclei with 4′,6-diamidino-2-phenylindole (DAPI, 1:1000, cat# D3571, Life Technologies, Carlsbad, CA). Apoptosis was assessed by staining with anti-cleaved caspase 3 (Cell Signaling, 1:200, cat #9661, Danvers, MA) or by terminal deoxynucleotidyl transferase dUTP nick-end labeling (TUNEL) assay (TACS 2Tdt-Fluor in situ apoptosis detection kit, Trevigen, cat# 4812-30-K, Gaithersburg, MD). A positive control for the TUNEL assay was included in the kit and performed according to the manufacturer’s directions. Secondary antibodies were either donkey anti-rabbit or goat anti-mouse conjugated to Alexa 555 (1:1000, Invitrogen, Waltham, MA) and were applied at room temperature (20–22 °C) for 2 h.

All immunohistochemical analyses were performed by a blinded researcher using ImageJ (NIH, Bethesda, MD). At least two sections per animal, at least five sections apart, were averaged for each antibody. Sections were imaged on a Nikon TE2000 microscope (Nikon, Melville, NY) with a nuance spectral camera (Quorum, model# N-MSI-FX, Guelph, Ontario), which allows for elimination of background and low-intensity fluorescence as previously described [41]. Images were taken at 20X and 60X for all but GFAP intensity, which was imaged at 10X and 60X. Lobules of the cerebellar vermis have been shown to have different sensitivities to PAE [42] likely based on their rates of maturation. Therefore, lobules were grouped into three regions for quantification; lobules I–III, IV–VII, and IX–X. The following hippocampal formation regions were analyzed: CA1, CA3, and dentate gyrus (DG).

For measurements of intensity, the region of interest was traced and fluorescent intensity was quantified within. GFAP intensity was used to assess astrocytes (reviewed by [43]).

Microglia were stained with IBA-1 and classified into one of four morphological stages, similar to as previously described [44, 45]. In brief, cell types were categorized as either resting/ramified (characterized by small cell body and long dendritic processes), transitional 1 (slightly swollen cell body with thicker, shorter processes), transitional 2 (large cell body with slight protrusion of processes), or amoeboid (large, round cell body with no processes and more intensely stained with IBA-1).

To quantify neuronal number, the thickness of the cell layer and/or the total cell number was assessed. In the case of cell layer thickness, measurements were made randomly in three separate locations and averaged per image. To quantify neuronal number, cells were counted using unbiased stereological techniques, based on the optical fractionator method, as previously described [46]. In brief, images were overlaid with an optical dissector grid system in which each grid contained a small box (counting frame) in the upper left-hand corner. Positively stained cells falling within the unbiased counting frame or touching the allowed edges of the boxes were included. Data are displayed as the average number of cells within the counting frames of multiple sections/images.

### Gait analysis

**Fig. 2:**
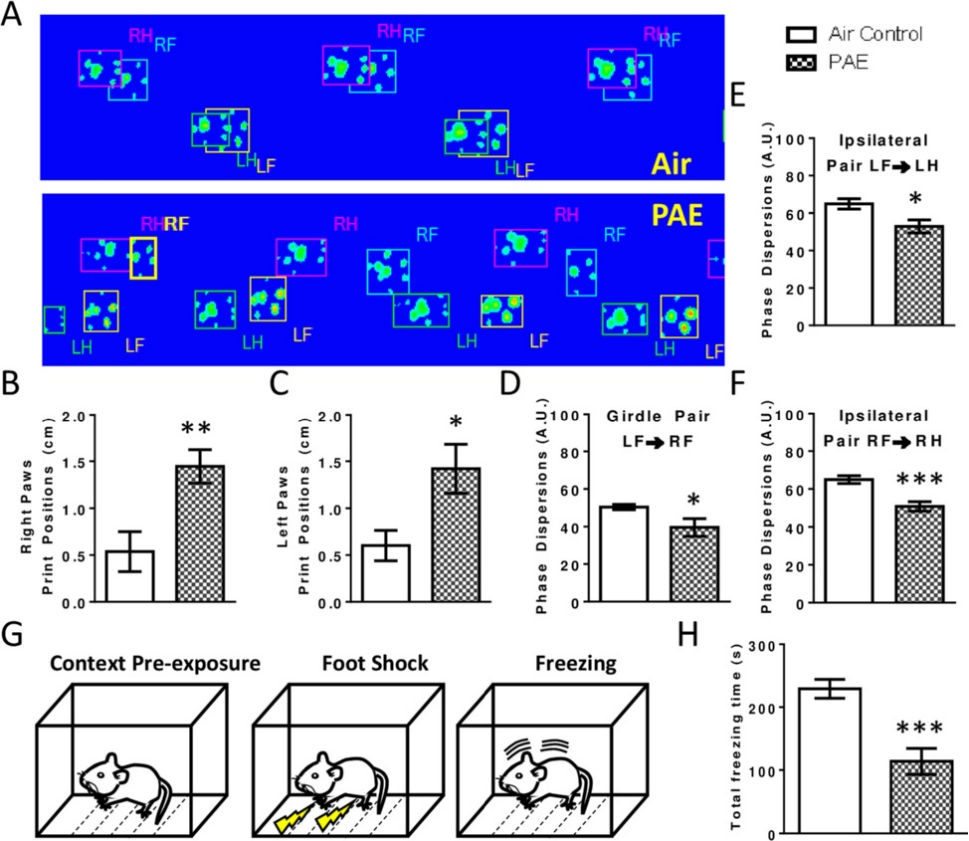
Postnatal alcohol exposure (PAE) induced alterations in gait and deficits in fear conditioning. **a**–**f** Animals aged P45–50 were placed on a Catwalk apparatus and differences in gait were assessed (*n* = 9 animals from 4 litters). **a** Representative Catwalk images are shown illustrating a PAE-induced reduction in overlap between the position of front and hind paws (*LF* left forepaw, *LH* left hindpaw, *RF* right forepaw, *RH* right hindpaw). Summary graphs illustrating the overall effect of PAE on the distance between placement of fore and hind paws on the right (**b**) and left (**c**) side of the body. **d**–**f** Phase dispersions, which are indicators of inter-limb coordination, were measured between paws. **g**–**h** Animals aged P36–48 (*n* = 5–7 animals from 5–7 litters) were tested for performance on a variation of the fear conditioning test in which they were pre-exposed to the context the day before being re-exposed to the context and then shocked. (*p < 0.05, **p < 0.01, ***p < 0.001)

Alterations in gait are associated with cerebellar impairment and particularly with Purkinje cell dysfunction [47–49]. Gait was assessed using the Catwalk XT system (Noldus, Wageningen, Netherlands) located at the Animal Behavioral Core of the Biomedical Research and Integrative Neuroimaging Center (BRaIN), UNM-HSC; two to three animals per litter were used from a total of 4 litters per treatment group. Rats aged P45–50 (Fig. 2a) were placed on an enclosed glass walkway illuminated from above by a red fluorescent light and along the walkway by green light-emitting diode lights. Disruption of the green light allowed for tracking of paw prints, while disruption of red light allowed for visualization of silhouettes. Rats were allowed to walk freely across the runway to a dark box, containing bedding from their home cage, located at the opposite end. A digital high-speed camera recorded each trial until three compliant trials were captured in which the animal did not stop or turn around and crossed the walkway within 0.5–10 s with a maximum body speed variation of less than 60 %. PAE significantly increased the total number of trials required to achieve three compliant trials, and these were 3.44 ± 0.41 and 6.44 ± 1.28 trials for the control and alcohol groups, respectively (t(_16_) = 2.23, *p* = 0.041, by unpaired *t* test). After each trial, the walkway was wiped down with 70 % ethanol and deionized water. Catwalk XT 8.1 software (Noldus) was used to analyze the data. A detailed description of the measured parameters can be found in Additional file 1. For each animal, print area, average print intensity, base of support, support percentage, print position, phase dispersions, swing speed, cadence, and average speed were calculated, similar to as previously described [50]. Parameters were analyzed for the right forepaw (RF), right hind paw (RH), left forepaw (LF), and left hind paw (LH).

### Contextual fear conditioning

Multiple pre-exposure contextual fear conditioning was used to test hippocampal-dependent behavioral differences. Pre-exposing animals to the context 1 day prior to shock conditioning, which has been shown to increase freezing, is impaired in a model of neonatal alcohol exposure [51]. Animals aged P36–48 (Fig. 1a) were individually housed 1 day prior to testing. One animal per litter from 5–7 litters per treatment group was used. Between animals, the chamber was cleaned with ~5 % ammonium chloride and left to dry for 2 min before the next animal was placed into the chamber. Testing took place over 3 days. On day 1, the animals were allowed to explore the chamber for 5 min. Then, the animals were removed from the chamber for 1 min and then returned to the chamber for the next minute. This was repeated a total of five times before returning the animal to the home cage. On the second day, animals were placed into the chamber and received a 2-s foot shock (1.5 mA) ~45–60 s later, after which they were placed back into their home cage. On the final day of testing, the animal was placed in the chamber for 5 min and total freezing time, defined as the body becoming immobile without any licking or grooming activity, was video-recorded, and then scored by a blinded observer.

### Statistical analysis

All statistics were analyzed with Prism Version 6.05 (GraphPad Software, San Diego, CA). Data were analyzed by student’s unpaired *t* test or two-way ANOVA followed by Bonferroni post hoc test. Significance was determined as *p* < 0.05. Data are shown as mean ± SEM.

## Results

### Characterization of the exposure paradigm

PAE significantly decreased pup body weight on P17 (Fig. 1b, two-way ANOVA, interaction F(_5,98_) = 4.00, *p* = 0.002; age F(_5, 98_) = 153.2, *p* < 0.0001; alcohol F(_1,98_) = 12.01, *p* = 0.0008); Bonferroni post hoc test: *p* = 0.0004 on P17). Nursing, as assessed by the proportion of pups with milk in their stomachs, was not significantly affected by PAE (Fig. 1c, two-way ANOVA, interaction F(_3,36_) = 2.44, *p* = 0.080; age F(_3,36_) = 2.15, *p* = 0.111; alcohol F(_1,36_) = 0.73, *p* = 0.400). Litter sizes were comparable between PAE and air control groups (Fig. 1d, t(_32_) = 1.25, *p* = 0.222, unpaired *t* test). In pups, BACs reached a peak of 121.1 ± 11.75 mM (0.56 ± 0.05 g/dL) at the end of exposure and returned to baseline by the following morning (Fig. 1e). Conversely, peak maternal BACs were much lower at 32.22 ± 8.49 mM (Fig. 1e). Measurements of maternal care were unaffected by alcohol exposure (Table 1).

**Table 1:** Assessment of maternal care

Characterization	Air (% of total observation time)	PAE (% of total observation time)	t	*p* value
No contact with pups	23.98 ± 4.05	12.50 ± 8.41	1.23	0.242
Licking pups and arched-back nursing	7.10 ± 2.16	3.95 ± 2.54	0.94	0.365
Arched-back nursing	29.86 ± 7.00	26.02 ± 9.51	0.33	0.751
Blanket nursing	23.90 ± 7.76	46.68 ± 11.18	1.67	0.120
Passive nursing	7.80 ± 2.67	5.66 ± 2.91	0.54	0.597
Licking pups	7.37 ± 1.24	5.19 ± 1.48	1.13	0.281

Maternal care was assessed hourly throughout exposure and for several hours following exposure (as described in the methods). Key maternal behaviors were scored and displayed above. DF = 12, *n* = 7

To determine if this PAE paradigm induced deficits in cerebellar-dependent behavior, rats were tested on a Catwalk apparatus and several parameters for gait were analyzed as described in Additional file 1. Alterations in gait are associated with cerebellar impairment and particularly with Purkinje cell dysfunction [47–49]. PAE had significant effects on many aspects of gait (Additional file 2, Fig. 2a-f), including phase dispersions between the LF → LH paws (Fig. 2e, t(_16_) = 2.67, *p* = 0.017, unpaired *t* test), the RF → RH paws (Fig. 2f, t(_16_) = 4.35, *p* = 0.0005, by unpaired *t* test), and the LF → RF paws (Fig. 2d, t(_16_) = 2.21, *p* = 0.042, unpaired *t* test), which are determinants of inter-limb coordination. Of note, print positions, on both the right (Fig. 2b, t(_16_) = 3.26, *p* = 0.005, by unpaired *t* test) and left (Fig. 2c, t(_16_) = 2.66, *p* = 0.017, by unpaired *t* test) sides of the body were affected after PAE. In a normal gait, the hind paw makes contact with the walkway in nearly the same location as the previous step of the forepaw on the same side of the body. In PAE animals, the distance between placement of these paws was noticeably increased (Fig. 2a). Spatial parameters related to individual paws such as print area and intensity were unaffected by PAE (Additional file 2). Additionally, while PAE decreased the average speed of the animal and the swing(s) of the LF, it had no other effects on temporal parameters (Additional file 2).

To determine if PAE elicited any deficits in hippocampal-dependent behavior, performance in the multiple pre-exposure contextual fear conditioning test was assessed (Fig. 2g). PAE animals spent significantly less time freezing in response to the context in which they had previously received a foot shock than control animals (Fig. 2h, *t*(_10_) = 4.64, *p* = 0.0009, unpaired *t* test).

### PAE decreases neuronal number in the cerebellar vermis

**Fig. 3:**
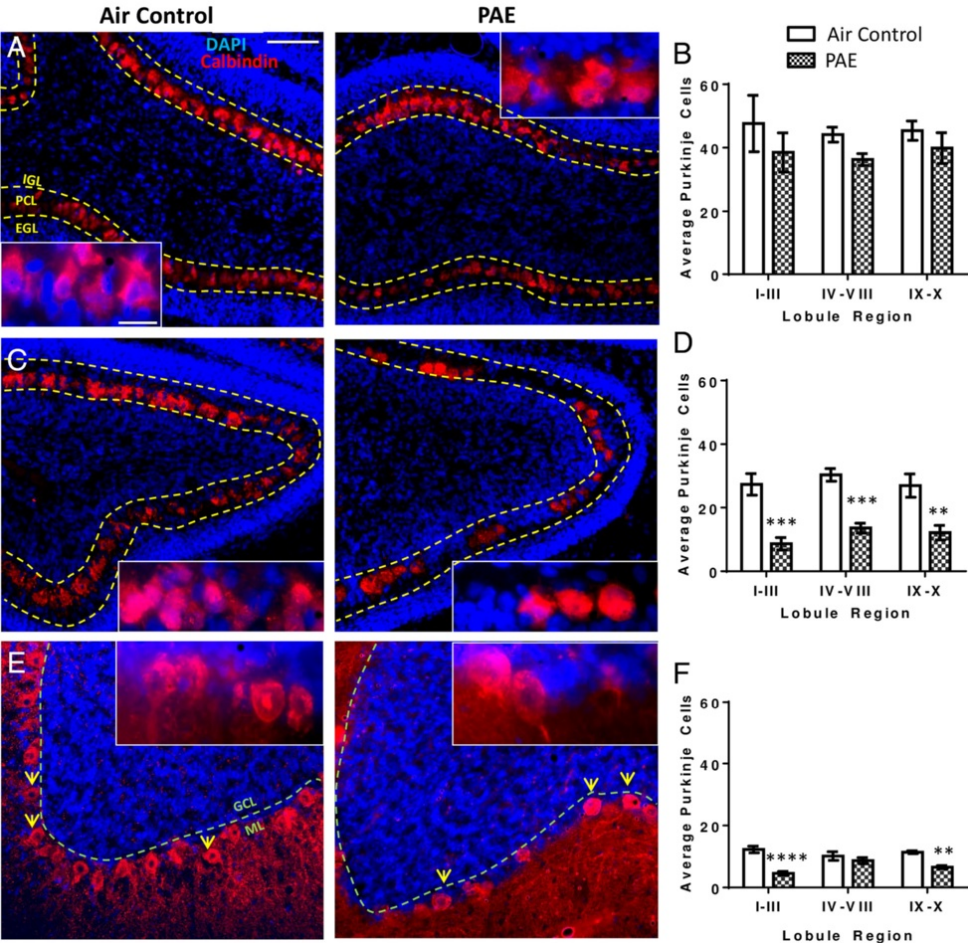
Postnatal alcohol exposure (PAE) reduced the number of Purkinje neurons in the cerebellar vermis. Representative images of the cerebellar vermis stained for calbindin (*red*) to label Purkinje neurons and 4′,6-diamidino-2-phenylindole (DAPI, *blue*) to label cell nuclei. Purkinje cell number was quantified during the first withdrawal period on P4 (**a**–**b**), the third withdrawal period on P6 (**c**–**d**) and also on P45 (**e**–**f**) (as shown in Fig. 1a). The cerebellar vermis was divided into three lobule regions and quantified separately. Purkinje cells were counted in each image and the average cells falling within the counting frame per image was averaged. (**p < 0.01, ***p < 0.001, ****p < 0.0001). *n* = 4 animals from 4 litters. The thickness of the external and internal granule cell layers (EGL and IGL, respectively) was also quantified and results are shown in Additional file 3. *Scale bars* are 40 and 10 μm for low and high magnification images, respectively

On the morning after the first exposure (i.e., first withdrawal period at P4; as shown in Fig. 1a), no effect of PAE was observed on Purkinje cell number across any of the lobule regions (Fig. 3, two-way ANOVA, interaction F(_2,18_) = 0.06, *p* = 0.938; lobule F(_2,18_) = 0.18, *p* = 0.836; alcohol F(_1,18_) = 3.17, *p* = 0.092). Similarly, thickness of the external granule layer (EGL) was unaffected by PAE (Additional file 3a, two-way ANOVA, interaction F(_2,18_) = 0.21, *p* = 0.816; lobule F(_2,18_) = 0.21, *p* = 0.031; alcohol F(_1,18_) = 0.39, *p* = 0.538) as well as the internal granule layer (IGL, Additional file 3b, two-way ANOVA, interaction F(_2,18_) = 0.20, *p* = 0.820; lobule F(_2,18_) = 2.32, *p* = 0.127; alcohol F(_1,18_) = 1.93, *p* = 0.182). However, at the end of exposure on P6, the alcohol exposed pups showed a dramatic reduction in the number of Purkinje neurons present in the cerebellar vermis in all lobule regions (Fig. 3c, d, two-way ANOVA, interaction F(_2,18_) = 0.27, *p* = 0.765; lobule F(_2,18_) = 60.01, *p* = 0.332; alcohol F(_1,18_) = 62.62, *p* < 0.0001; Bonferroni post hoc test: I-III, *p* = 0.0002; IV–VIII, *p* = 0.0007; IX-X, *p* = 0.002). There was also an overall decrease in thickness of the EGL (Additional file 3c, two-way ANOVA, interaction F(_2,18_) = 0.26, *p* = 0.776; lobule F(_2,18_) = 0.66, *p* = 0.527; alcohol F(_1,18_) = 7.87, *p* = 0.012), although there was no significant effect in individual lobule regions as measured by post hoc analysis. Furthermore, IGL thickness was not affected (Additional file 3d, two-way ANOVA, interaction F(_2,18_) = 0.37, *p* = 0.694; lobule F(_2,18_) = 1.101, *p* = 0.354; alcohol F(_1,18_) = 0.01, *p* = 0.912).

On P45, the number of Purkinje cells continued to be decreased in lobule regions I–III and IX–X, but not in region IV–VII (Fig. 3e, f, two-way ANOVA, interaction F(_2,18_) = 5.94, *p* = 0.010; lobule F(_2,18_) = 0.61, *p* = 0.554; alcohol F(_1,18_) = 40.37, *p* < 0.0001; Bonferroni post hoc test: I-III *p* < 0.0001, IX–X *p* = 0.004). These findings are consistent with previous studies showing lobules IV–VIII to be less sensitive to PAE [42]. Interestingly, granule cell layer thickness was no longer affected at P45 (Additional file 3e, two-way ANOVA, interaction F(_2,18_) = 1.16, *p* = 0.337; lobule F(_2,18_) = 1.664, *p* = 0.217; alcohol F(_1,18_) = 0.08, *p* = 0.774), indicating that PAE induces long-term selective loss of Purkinje neurons.

### PAE does not induce apoptosis in the cerebellar vermis during the first withdrawal period at P4

Since we detected a significant reduction in Purkinje cell number at P6, we investigated whether this was a consequence of activation of apoptotic pathways at an earlier time point. To accomplish this, we first stained sections collected during the first withdrawal period at P4 (Fig. 1a) for cleaved (activated) caspase 3 (Additional file 4). Consistent with previous studies showing that activated caspase 3 is expressed normally during development in the cerebellum and that it can have non-apoptotic functions in neurons [52–54], we detected staining for this protein in the air controls (Additional file 4a). However, we observed little effect of PAE on activated caspase 3 in the cerebellar vermis of P4 animals. There was a significant overall increase in caspase 3 expression in the EGL but not by post hoc analysis (Additional file 4b, two-way ANOVA, interaction F(_2.18_) = 0.04, *p* = 0.963; lobule F(_2,18_) = 0.84, *p* = 0.448; alcohol F(_1,18_) = 4.42, *p* = 0.0498). Caspase 3 expression was not significantly affected in the Purkinje cell layer (PCL) (Additional file 4b, two-way ANOVA, interaction F(_2.18_) = 0.07, *p* = 0.929; lobule F(_2,18_) = 0.75, *p* = 0.486; alcohol F(_1,18_) = 2.62, *p* = 0.123), or the IGL (Additional file 4b, two-way ANOVA, interaction F(_2.18_) = 0.14, *p* = 0.872; lobule F(_2,18_) = 1.10, *p* = 0.354; alcohol F(_1,18_) = 3.00, *p* = 0.100).

Since caspase 3 has been shown to have non-apoptotic roles in the cerebellum during development, we also assessed for apoptosis by TUNEL assay on P4 (Additional file 5). We found no difference between air control and PAE animals in the EGL (Additional file 5c, two-way ANOVA, interaction F(_2,17_) = 0.13, *p* = 0.875; lobule F(_2,17_) = 0.00, *p* = 0.999; alcohol F(_1,17_) = 0.00, *p* = 0.983), the PCL (Additional file 5c, two-way ANOVA, interaction F(_2,17_) = 0.19, *p* = 0.828; lobule F(_2,17_) = 0.07, *p* = 0.936; alcohol F(_1,17_) = 0.06, *p* = 0.804), or the IGL (Additional file 5c, two-way ANOVA, interaction F(_2,17_) = 0.17, *p* = 0.846; lobule F(_2,17_) = 0.37, *p* = 0.697; alcohol F(_1,17_) = 0.17, *p* = 0.682). Bonferroni’s post hoc test did not detect a significant effect of PAE in any of these cerebellar layers.

### PAE increases cytokine expression in the cerebellar vermis during periods of withdrawal

**Fig. 4:**
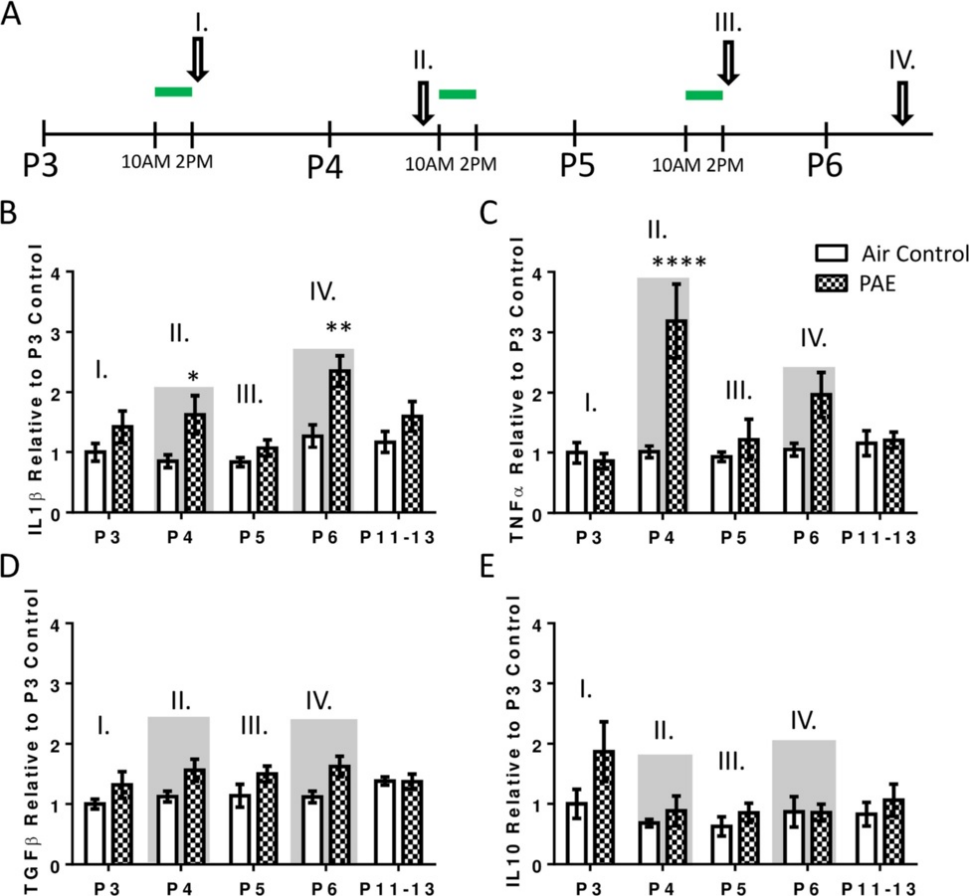
Postnatal alcohol exposure (PAE) increases cytokine expression in the cerebellar vermis. **a** Animals were exposed to alcohol from P3–5 for 4 h daily (10 a.m. to 2 p.m., represented by *green lines*). Expression of cytokines was measured by RT-PCR throughout the exposure. Time points during peak blood alcohol levels were collected on P3 (I) and P5 (III). Time points during withdrawal periods were collected on P4 (II) and P6 (IV) and are also indicated by *gray shading* in panels **b**–**e**. Effect of PAE on interleukin-1β (IL-1β, **b**), tumor necrosis factor α (TNFα, **c**), transforming growth factor β (TGFβ, **d**), and interleukin-10 (IL10, **e**) in the cerebellar vermis. For more details on the time course of the blood alcohol concentrations, please see Fig 1e. To address if effects were persistent, P11–13 was also included. Threshold cycle values (CT) were normalized to hypoxanthine phosphoribosyltransferase 1(HPRT1), and each individual value was normalized with respect to the average of the P3 air controls (see the “Materials and Methods” section). (*p < 0.05, **p < 0.01, ****p < 0.0001). *n* = 8 animals from 8 litters

To determine how the neuroimmune response evolves as a function of multiple exposures, pro- and anti-inflammatory cytokine responses were measured by reverse transcriptase polymerase chain reaction (RT-PCR) throughout the exposure and after it was completed (Fig. 4a). PAE increased the expression of pro-inflammatory IL-1β during withdrawal periods, which were collected on P4 and P6 (Fig. 4b, two-way ANOVA, interaction F(_4,70_) = 1.34, *p* = 0.265; age F(_4,70_) = 4.72, *p* = 0.002; alcohol F(_1,70_) = 20.65, *p* < 0.0001; Bonferroni post hoc test for PAE: P4 *p* = 0.045; P6 *p* = 0.002) but not significantly on time points collected during peak BACs (P3 and P5; Fig 4a). Similarly, TNFα expression was increased during the first withdrawal period on P4 (Fig. 4c, two-way ANOVA, interaction F(_4,70_) = 5.70, *p* = 0.0005; age F(_4,70_) = 5.66, *p* = 0.0005; alcohol F(_1,70_) = 14.06, *p* = 0.0004; Bonferroni post hoc test for PAE: P4 *p* < 0.0001). Additionally, PAE significantly increased overall expression of anti-inflammatory cytokine TGFβ (Fig. 4d, two-way ANOVA, interaction F(_4,69_) = 0.95, *p* = 0.442; age F(_4,69_) = 0.73, *p* = 0.577; alcohol F(_1,69_) = 12.24, *p* = 0.0008). However, expression was not found to be significantly elevated by post hoc test on any individual postnatal day. Additionally, expression of the anti-inflammatory cytokine IL10 was not affected by alcohol (Fig. 4e, two-way ANOVA, interaction F(_4,69_) = 0.89, *p* = 0.475; age F(_4,69_) = 2.53, *p* = 0.048; alcohol F(_1,69_) = 3.70, *p* = 0.059; one outlier was removed from the air control on P5 as determined by ROUT method).

### PAE induces morphological changes in microglia and increases astrocytic GFAP expression in the cerebellar vermis

**Fig. 5:**
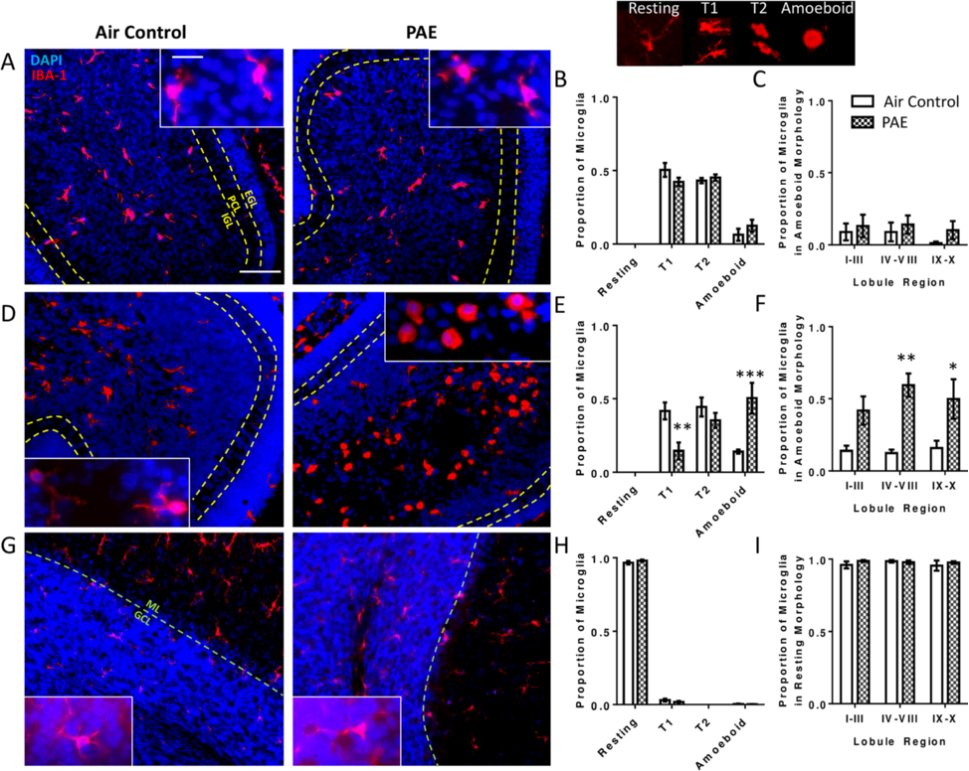
Postnatal alcohol exposure (PAE) activates microglia in the cerebellar vermis. Representative images of the cerebellar vermis are stained for ionized calcium-binding adapter protein molecule 1 (IBA-1, *red*) to label microglia and 4′,6-diamidino-2-phenylindole (DAPI, *blue*) to label cell nuclei. Microglial morphology was quantified as resting, transitional 1 (T1), transitional 2 (T2), or amoeboid as shown in the *top right panel*. The proportion of microglia exhibiting each morphology was quantified during the first withdrawal period on P4 (**a**–**b**), the third withdrawal period on P6 (**d**–**e**), and also on P45 (**g**–**h**) (as shown in Fig. 1a). To investigate regional differences, the cerebellar vermis was divided into three lobule regions and the proportion of microglia in an amoeboid morphology was quantified in each region on P4 (**c**) and P6 (**f**). On P45 (**i**), the proportion of resting microglia was quantified by region as there were no amoeboid microglia. (*p < 0.05, **p < 0.01, ***p < 0.001). *n* = 4 animals from 4 litters. *Scale bars* are 40 μm and 10 μm for representative low and high magnification images, respectively. The external granule cell (EGL), Purkinje cell (PCL), and internal granule cell (IGL) layers are labeled in panels *a* and *g*

To determine if the increase in cytokine levels is associated with alterations in glial cells, we stained for the microglia marker IBA-1 and the astrocyte marker GFAP. During the first withdrawal period (P4; Fig. 1a), the majority of microglia existed in a transitional state, with none exhibiting a ramified/resting morphology. However, microglia in PAE animals appeared very similar to those in controls (Fig. 5a–c). There was no interaction between the proportion of microglia in each morphological state and PAE (Fig. 5b, two-way ANOVA, interaction F(_3,24_) = 2.0, *p* = 0.141; morphology F(_3,24_) = 124.2, *p* < 0.0001; alcohol F(_1,24_) = 9.17e-12, *p* > 0.9999), and the same result was found across lobule regions (Fig. 5c, two-way ANOVA, interaction F(_2,18_) = 0.09, *p* = 0.911; lobule F(_2,18_) = 0.57, *p* = 0.574; alcohol F(_1,18_) = 1.53, *p* = 0.232).

By the end of the exposure paradigm, during the third withdrawal period (P6; Fig. 1a), there was a significant interaction between the proportion of microglia in each morphological state and PAE; i.e., while most microglia in control animals continued to exhibit a transitional morphology, PAE animals had an elevated presence of amoeboid microglia in the cerebellar vermis and a concurrent decrease in the number of microglia in the transitional 1 (T1) morphology (Fig. 5d, e, two-way ANOVA, interaction F(_3,24_) = 11.69, *p* < 0.0001; morphology F(_3,24_) = 19.69, *p* < 0.0001; alcohol F(_1,24_) = 1.81e-13, *p* > 0.999; Bonferroni post hoc test: air control versus PAE in amoeboid cells *p* = 0.0004 and in T1 cells *p* = 0.008). Similarly, the proportion of microglia in the amoeboid morphology was significantly increased by PAE in lobule regions IV–VIII and IX–X but not in lobule region I–III (Fig. 5f, two-way ANOVA, interaction F(_2,18_) = 0.73, *p* = 0.496; lobule F(_2,18_) = 0.51, *p* = 0.609; alcohol F(_1,18_) = 30.24, *p* < 0.0001; Bonferroni post hoc test: IV-VIII *p* = 0.002; IX–X *p* = 0.024). To investigate if changes in microglial morphology persist, staining was also assessed on P45 (Fig. 1a). By this age, nearly all microglia in both PAE and control animals existed in a resting state and there was no interaction between treatment and morphology (Fig. 5g, h, two-way ANOVA, interaction F(_3,24_) = 1.33, *p* = 0.289; morphology F(_3,24_) = 10612, *p* < 0.0001; alcohol F(_1,24_) = 1.87e-10, *p* > 0.999). Similarly, there was no effect of PAE on the proportion of microglia in a resting morphology across lobule regions (Fig. 5i, two-way ANOVA, interaction F(_2,18_) = 0.47, *p* = 0.633; lobule F(_2,18_) = 0.36, *p* = 0.706; alcohol F(_1,18_) = 0.79, *p* = 0.385).

**Fig. 6:**
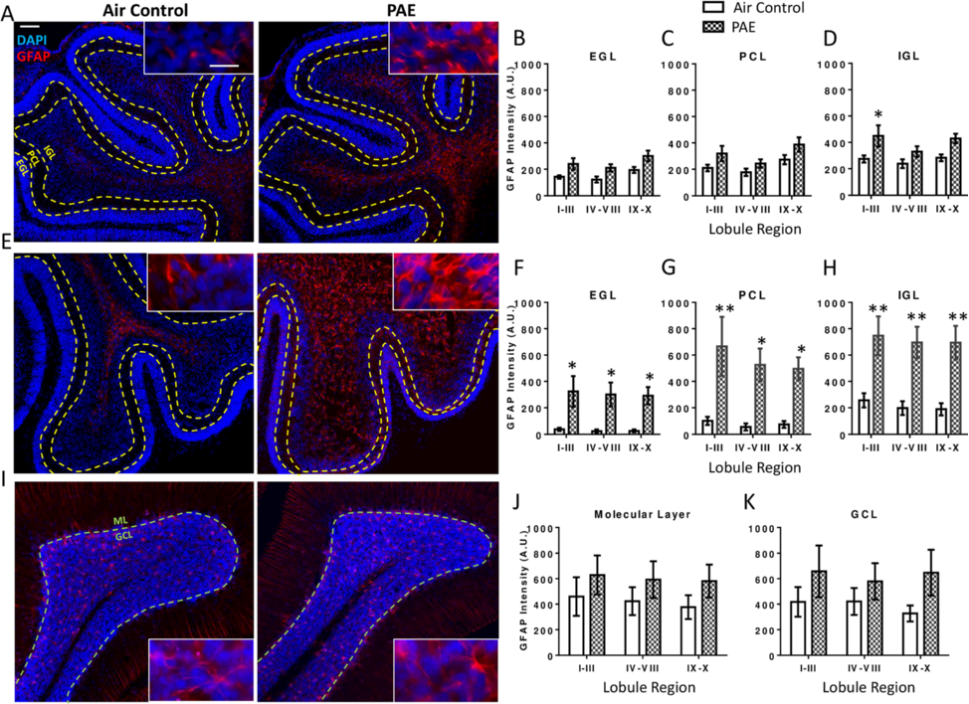
Postnatal alcohol exposure (PAE) increases astrocytic glial fibrillary acidic protein (GFAP) expression in the cerebellar vermis. Representative images of the cerebellar vermis are stained for GFAP (*red*) to label astrocytes and 4′,6-diamidino-2-phenylindole (DAPI, *blue*) to label cell nuclei. The cerebellar vermis was divided into three lobule regions and quantified separately during the first withdrawal period on P4 (**a**–**d**), the third withdrawal period on P6 (**e**–**h**), and also on P45 (**i**-**k**) (as shown in Fig. 1a). On P4 and P6, the external granule layer (EGL), Purkinje cell layer (PCL), and internal granule layer (IGL) of each lobule were traced as shown. On P45 the molecular layer (ML) and granule cell layer (GCL) of each lobule were traced (the EGL is no longer present at this age). GFAP intensity levels were measured within each layer. (*p < 0.05, **p < 0.01). *n* = 4 animals from 4 litters. *Scale bars* are 40 and 10 μm for low and high magnification images, respectively

To examine astrocytes, changes in GFAP intensity were measured in the EGL, PCL, and IGL of the lobules. During the first withdrawal period (P4), PAE caused an overall significant increase in GFAP expression in the EGL (Fig. 6a, b, two-way ANOVA, interaction F(_2,18_) = 0.05, *p* = 0.954; lobule F(_2, 18_) = 3.70, *p* = 0.045; alcohol F(_1,18_) = 15.39, *p* = 0.001) and the PCL (Fig. 6a, c, two-way ANOVA, interaction F(_2,18_) = 0.21, *p* = 0.817; lobule F(_2,18_) = 4.34, *p* = 0.029; alcohol F(_1,18_) = 8.55, *p* = 0.009) of the cerebellar vermis but not in any individual lobule region by post hoc test. In the IGL, GFAP expression was significantly increased in lobule regions I–III (Fig. 6a, d, two-way ANOVA, interaction F(_2,18_) = 0.44, *p* = 0.649; lobule F(_2,18_) = 1.87, *p* = 0.182; alcohol F(_1,18_) = 14.21, *p* = 0.001; Bonferroni post hoc test: *p* = 0.039).

By the end of the exposure paradigm, during the third withdrawal period (P6), post hoc analysis showed that PAE increased astrocytic GFAP expression across all three lobule regions in the EGL (Fig. 6e, f, two-way ANOVA, interaction F(_2,18_) = 0.01, *p* = 0.987; lobule F(_2,18_) = 0.06, *p* = 0.938; alcohol F(_1,18_) = 25.61, *p* < 0.0001; Bonferroni post hoc test: I–III *p* = 0.022; IV–VIII *p* = 0.027; IX–X *p* = 0.035), the PCL (Fig. 6e, g, two-way ANOVA, interaction F(_2,18_) = 0.21, *p* = 0.812; lobule F(_2,18_) = 0.49, *p* = 0.620; alcohol F(_1,18_) = 27.97, *p* < 0.0001; Bonferroni post hoc test: I–III *p* = 0.007; IV–VIII *p* = 0.025; IX–X *p* = 0.049), and in the IGL (Fig. 6e, h, two-way ANOVA, interaction F(_2,18_) = 0.00, *p* = 0.998; lobule F(_2,18_) = 0.22, *p* = 0.803; alcohol F(_1,18_) = 37.33, *p* < 0.0001; Bonferroni post hoc test: I–III *p* = 0.008; IV–VIII *p* = 0.007; IX–X *p* = 0.007). On P45, PAE no longer increased GFAP expression in either the ML (Fig. 6i, j, two-way ANOVA, interaction F(_2,18_) = 0.01, *p* = 0.988; lobule F(_2,18_) = 0.12, *p* = 0.884; alcohol F(_1,18_) = 2.83, *p* = 0.110) or the GCL (Fig. 6i, k, two-way ANOVA, interaction F(_2,18_) = 0.16, *p* = 0.852; lobule F(_2,18_) = 0.07, *p* = 0.934; alcohol F(_1,18_) = 4.19, *p* = 0.056).

### PAE does not reduce the number of granule cells or pyramidal neurons in the hippocampal formation

**Fig. 7:**
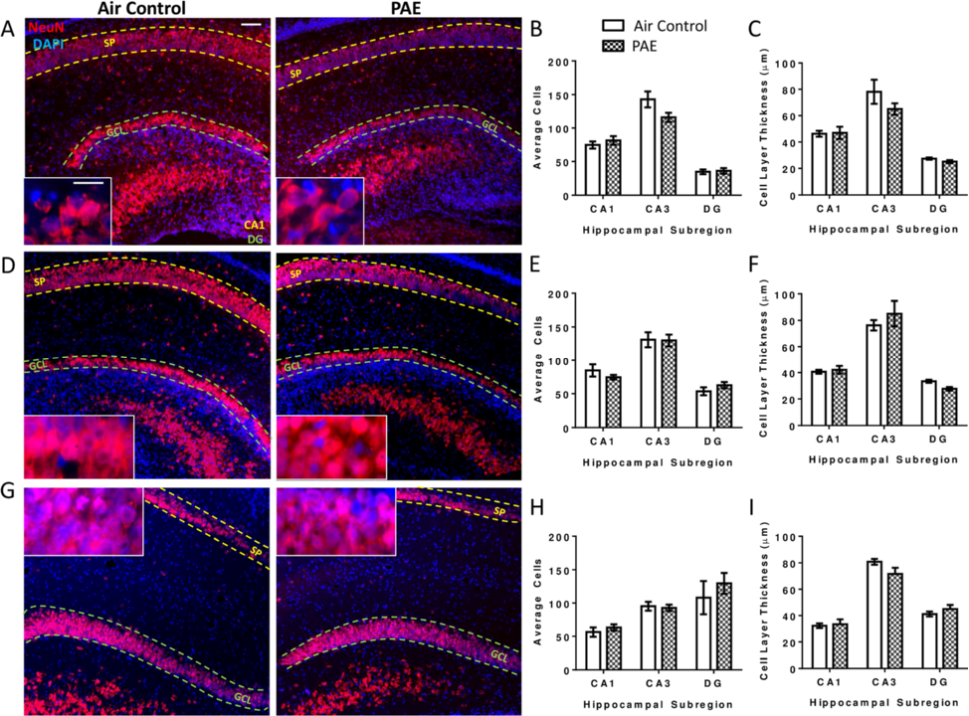
Postnatal alcohol exposure (PAE) does not alter the number of pyramidal neurons in the hippocampus. Representative images of the hippocampus are stained for NeuN (*red*) to label neurons and 4′,6-diamidino-2-phenylindole (DAPI, *blue*) to label cell nuclei during the first withdrawal period on P4 (**a**–**c**), the third withdrawal period on P6 (**d**–**f**) and also on P45 (**g**–**i**) (as shown in Fig. 1a). Cells were counted within the counting frame of each image in the *stratum pyramidale* (SP*)* layer of the CA3 and CA1, and in the granule cell layer (GCL) of the dentate gyrus (DG) subregion and averaged across sections. Thickness of the cell layers was also measured. *n* = 4 animals from 4 litters. *Scale bars* are 40 and 10 μm for representative images and high magnification pictures, respectively

To investigate potential regional differences in the effects of PAE, the hippocampal formation was also analyzed for neuronal loss in the GCL of the DG and in the pyramidal cell layer of the CA1 and CA3 subregions. During the first withdrawal period (P4), PAE animals exhibited no difference in neuronal number within the respective subregions (Fig. 7a, b, two-way ANOVA, interaction F(_2,18_) = 3.40, *p* = 0.056; region F(_2,18_) = 92.67, *p* < 0.0001; alcohol F(_1,18_) = 1.24, *p* = 0.280) as well as similar cell layer thicknesses (Fig. 7a, c, two-way ANOVA, interaction F(_2,18_) = 1.21, *p* = 0.322; region F(_2,18_) = 47.25, *p* < 0.0001; alcohol F(_1,18_) = 1.66, *p* = 0.214). Additionally, at the end of the exposure paradigm, during the third withdrawal period (P6), PAE animals showed no change in neuronal number in any subregion (Fig. 7d, e, two-way ANOVA, interaction F(_2,18_) = 0.79, *p* = 0.467; region F(_2,18_) = 47.28, *p* < 0.0001; alcohol F(_1,18_) = 0.01, *p* = 0.911) nor were there any differences in cell layer thicknesses (Fig. 7d, f, two-way ANOVA, interaction F(_2,18_) = 1.30, *p* = 0.297; region F(_2,18_) = 68.30, p < 0.0001; alcohol F(_1,18_) = 0.17, *p* = 0.685).

Similarly, on P45, there was no significant change in either neuronal number (Fig. 7g, h, two-way ANOVA, interaction F(_2,18_) = 0.43, *p* = 0.655; region F(_2,18_) = 10.42, *p* = 0.001; alcohol F(_1,18_) = 0.66, *p* = 0.427) or cell layer thicknesses (Fig. 7g, i, two-way ANOVA, interaction F(_2,18_) = 2.48, *p* = 0.112; region F(_2,18_) = 109.2, *p* < 0.0001; alcohol F(_1,18_) = 0.32, *p* = 0.579).

### PAE increases cytokine expression in the hippocampus

**Fig. 8:**
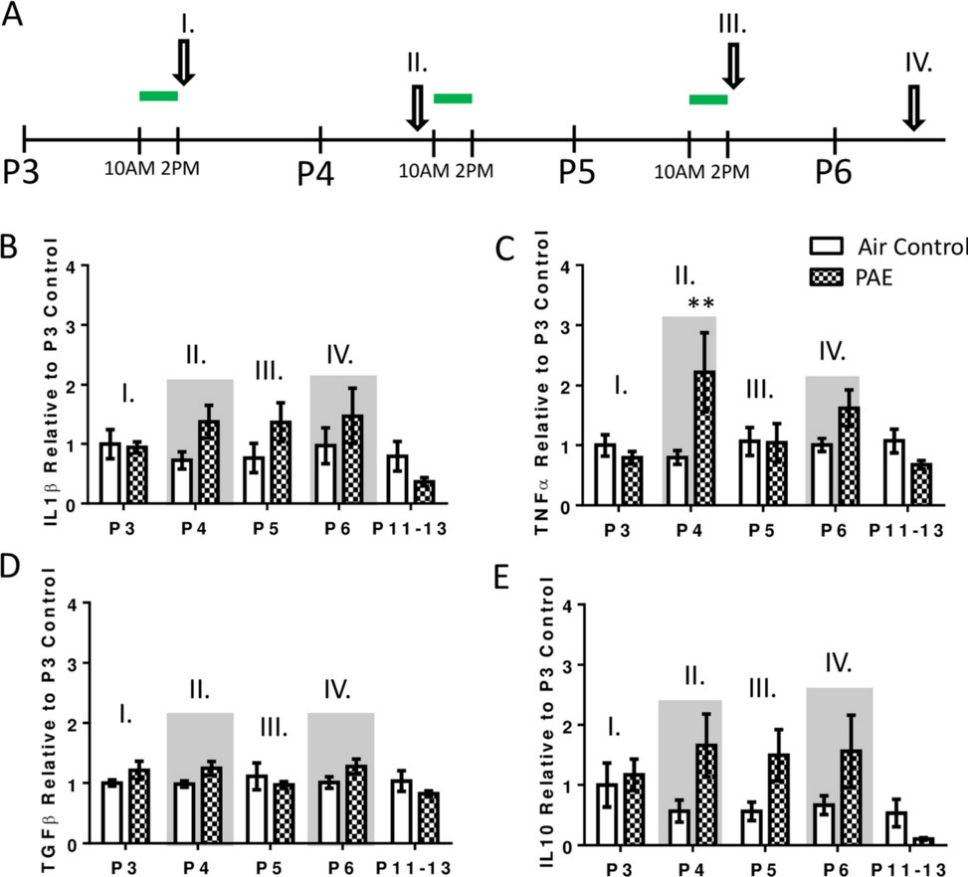
Postnatal alcohol exposure (PAE) increases cytokine expression in the hippocampus. **a**. Animals were exposed to alcohol from P3–5 for 4 h daily (10 a.m. to 2 p.m., represented by *green lines*). Expression of cytokines was measured by RT-PCR throughout the exposure. Time points during peak blood alcohol levels were collected on P3 (I) and P5 (III). Time points during withdrawal periods were collected on P4 (II) and P6 (IV) and are also indicated by *gray shading* in panels **b**–**e**. Effect of PAE on interleukin-1β (IL-1β, **b**), tumor necrosis factor α (TNFα, **c**), transforming growth factor β (TGFβ, **d**), and interleukin-10 (IL10, **e**) in the hippocampus. For more details on the timecourse of the blood alcohol concentrations, please see Fig. 1e. To address if effects were persistent, P11–13 was also included. Threshold cycle values (CT) were normalized to hypoxanthine phosphoribosyltransferase 1(HPRT1), and each individual value was normalized with respect to the average of the P3 air controls (see Materials and Methods). (**p < 0.01). *n* = 8 animals from 8 litters

To compare the neuroimmune response in the hippocampus with the cerebellar vermis, pro- and anti-inflammatory cytokines were measured by RT-PCR throughout and after the exposure (Fig. 8a). PAE had no effect on the expression of pro-inflammatory IL-1β (Fig. 8b, two-way ANOVA, interaction F(_4,69_) = 1.55, *p* = 0.197; age F(_4,69_) = 1.61, *p* = 0.183; alcohol F(_1,69_) = 2.21, *p* = 0.142). Conversely, there was a significant interaction between PAE and TNFα expression, and post hoc analysis revealed an increase during the first withdrawal period on P4 (Fig. 8c, two-way ANOVA, interaction F(_4,69_) = 3.29, *p* = 0.016; age F(_4,69_) = 1.82, *p* = 0.136; alcohol F(_1,69_) = 2.44, *p* = 0.123; Bonferroni post hoc test: P4 *p* = 0.005). Consistent with findings in the cerebellum, TNFα expression was not significantly elevated by post hoc analysis the following day during peak BAC (P5). PAE did not affect expression of anti-inflammatory TGFβ (Fig. 8d, two-way ANOVA, interaction F(_4,69_) = 1.78, *p* = 0.142; age F(_4,69_) = 0.97, *p* = 0.431; alcohol F(_1,69_) = 0.95, *p* = 0.334). In contrast, PAE increased overall expression of anti-inflammatory IL10 (Fig. 8e, two-way ANOVA, interaction F(_4,69_) = 1.76, *p* = 0.147; age F(_4,69_) = 2.03, *p* = 0.100; alcohol F(_1,69_) = 6.00, *p* = 0.017) but not on any individual day as assessed by post hoc analysis.

### PAE increases astrocytic GFAP expression in the hippocampus

**Fig. 9:**
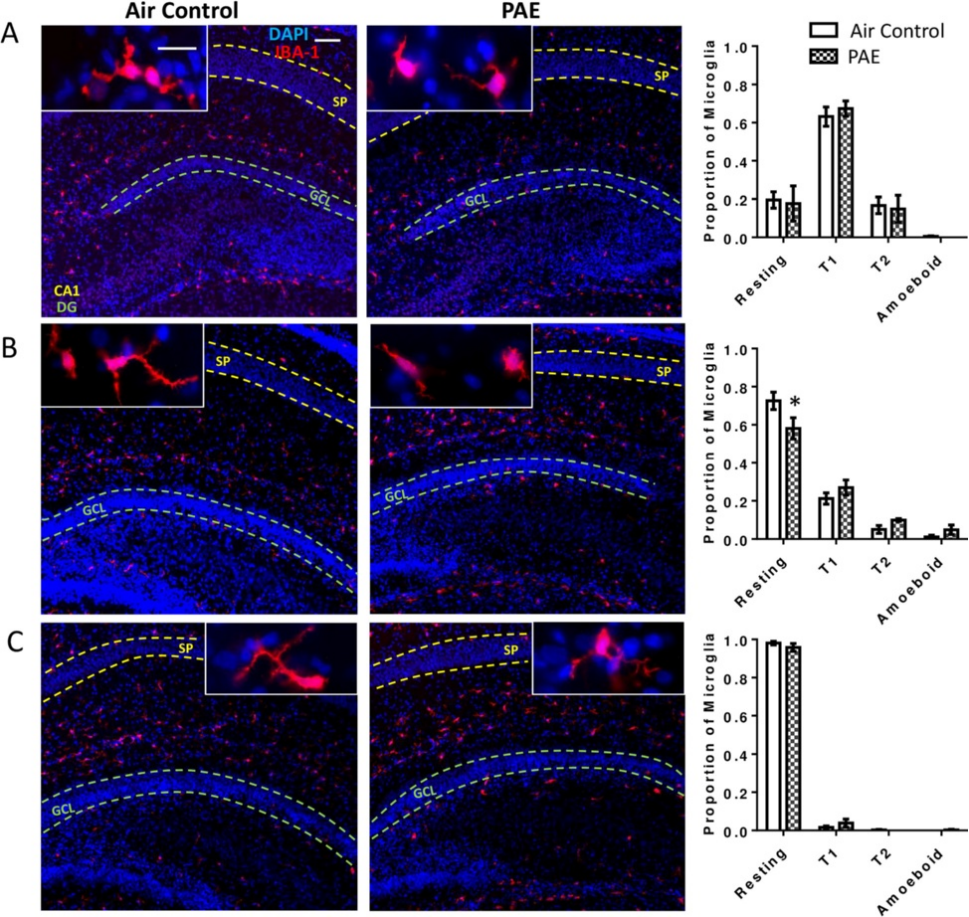
Postnatal alcohol exposure (PAE) decreases resting microglia on P6. The effect of PAE on microglial morphology in the hippocampus during the first withdrawal period on P4 (**a**), the third withdrawal period on P6 (**b**), and also on P45 (**c**) (as shown in Fig. 1a). Representative images of the hippocampus are stained for ionized calcium-binding adapter protein molecule 1(IBA-1, *red*) to label microglia and 4′,6-diamidino-2-phenylindole (DAPI, *blue*) to label cell nuclei. Microglial morphology was quantified as resting, transitional 1 (T1), transitional 2 (T2), or amoeboid as shown in Fig. 5. The proportion of microglia exhibiting each morphology was quantified. Since no significant differences were found in activation, morphology by region was not analyzed. *Asterisks* represent significance by post hoc test. (*p < 0.05). *n* = 4 animals from 4 litters. *Scale bars* are 40 and 10 μm for low and high magnification images, respectively. *Stratum pyramidale* (SP*)* of the CA1 region and the granule cell layer (GCL) of the dentate gyrus (DG) are labeled in the images

To assess morphological changes in glial cells in the hippocampus, astrocytes and microglia were stained with GFAP and IBA-1, respectively. During the first withdrawal period (P4), the majority of microglia exhibited a T1 morphology and there was no interaction between PAE and morphology (Fig. 9a, two-way ANOVA, interaction F(_3,24_) = 0.16, *p* = 0.924; morphology F(_3,24_) = 59.46, *p* < 0.0001; alcohol F(_1,24_) = 0.0, *p* > 0.999). By the third withdrawal period (P6), the majority of microglia in both groups exhibited a more mature, resting/ramified morphology, but there was a significant interaction between PAE and morphology, and post hoc analysis revealed a decrease in the proportion of microglia in a resting morphology (Fig. 9b, two-way ANOVA, interaction F(_3,24_) = 4.21, *p* = 0.016; morphology F(_3,24_) = 145.7, *p* < 0.0001; alcohol F(_1,24_) = 2.87e-11, *p* > 0.999; Bonferroni post hoc test: air control versus PAE in resting cells *p* = 0.021). However, PAE did not significantly increase the number of microglia in T1, T2, or amoeboid forms. Finally, on P45, no differences in microglial morphology were evident in the hippocampus of PAE animals (Fig. 9c, two-way ANOVA, interaction F(_3,24_) = 1.31, *p* = 0.295; morphology F(_3,24_) = 35, *p* < 0.0001; alcohol F(_1,24_) = 0.0, *p* > 0.999) and nearly all microglia in both groups were in a resting morphological state.

**Fig. 10:**
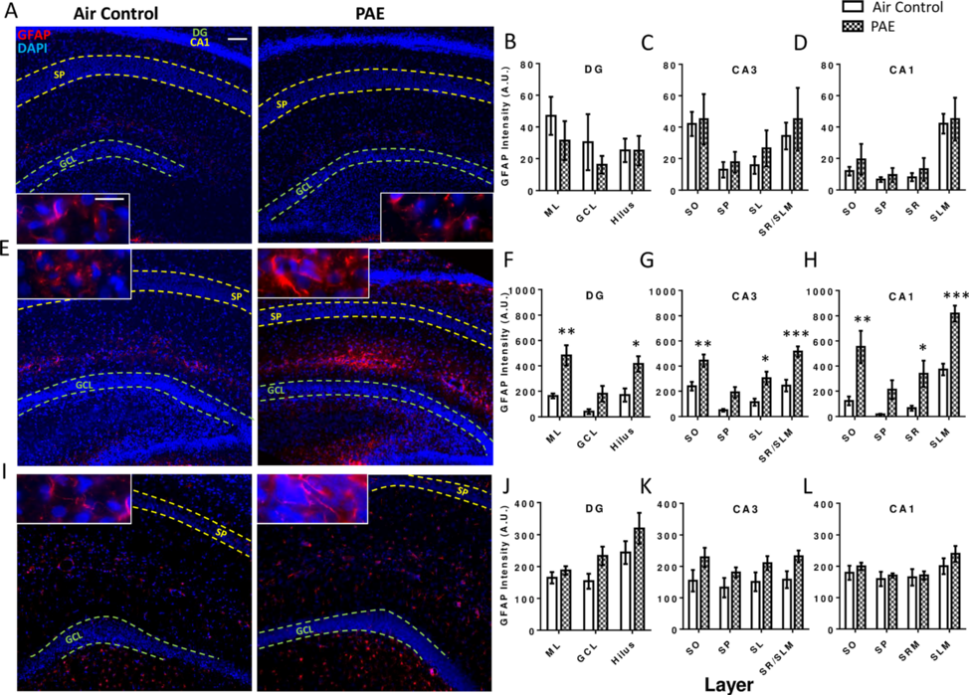
Postnatal alcohol exposure (PAE) increases astrocytic glial fibrillary acidic protein (GFAP) expression in the hippocampus. Representative images of the hippocampus are stained for GFAP (*red*) to label astrocytes and 4′,6-diamidino-2-phenylindole (DAPI, *blue*) to label cell nuclei during the first withdrawal period on P4 (**a**–**d**), the third withdrawal period on P6 (**e**–**h**), and also on P45 (**i**–**l**) (as shown in Fig. 1a). The CA1 and CA3 subregions the *stratum oriens* (SO), *stratum pyramidale* (SP) and either the *stratum radiatum* (SR), and *stratum lacunosum moleculare* (SLM) layers or the *stratum lucidum* (SL), respectively, were traced and GFAP intensity was quantified within. In the dentate gyrus (DG), the molecular layer (ML), granule cell layer (GCL), and hilus were quantified. (*p < 0.05, **p < 0.01, ***p < 0.001). *n* = 4 animals from 4 litters. *Scale bars* are 40 and 10 μm for low and high magnification images, respectively. *SP* of the CA1 region and the GCL of the DG are labeled in the images

In the CA1 and CA3 subregions, changes in GFAP intensity were quantified within the *stratum oriens* (SO), stratum pyramidale (SP), and either within the *stratum radiatum* (SR) and *stratum lacunosum moleculare* (SLM) or the *stratum lucidum* (SL) and SR/SLM, respectively. Similarly, the molecular layer (ML), GCL, and hilus were traced in the DG subregion and GFAP intensity was quantified. During the first withdrawal period (P4), PAE did not increase GFAP expression in the DG (Fig. 10a, b, two-way ANOVA, interaction F(_2,18_) = 0.28, *p* = 0.760; layer F(_2,18_) = 1.17, *p* = 0.334; alcohol F(_1,18_) = 1.16, *p* = 0.295), CA3 (Fig. 10a,c, two-way ANOVA, interaction F(_3,24_) = 0.06, *p* = 0.979; layer F(_3,24_) = 2.99, *p* = 0.051; alcohol F(_1,24_) = 0.83, *p* = 0.371) or the CA1 subregion (Fig. 10a, d, two-way ANOVA, interaction F(_3,24_) = 0.05, *p* = 0.986; layer F(_3,24_) = 10.51, *p* = 0.0001; alcohol F(_1,24_) = 0.81, *p* = 0.378).

By the third withdrawal period on P6, astrocyte activation was evident in the DG subregion in the ML and the hilus (Fig. 10e, f, two-way ANOVA, interaction F(_2,18_) = 1.42, *p* = 0.267; layer F(_2,18_) = 9.03, *p* = 0.002; alcohol F(_1,18_) = 28.42, *p* < 0.0001; Bonferroni post hoc test: ML *p* = 0.002; hilus *p* = 0.014). Similarly, the CA3 subregion exhibited increased GFAP expression in the SO, SL, and SR/SLM layers (Fig. 10e, g, two-way ANOVA, interaction F(_3,24_) = 0.89, *p* = 0.460; layer F(_3,24_) = 17.76, *p* < 0.0001; alcohol F(_1,24_) = 49.81, *p* < 0.0001; Bonferroni post hoc test: SO *p* = 0.006; SL *p* = 0.011; SR/SLM *p* = 0.0003). Additionally, in the CA1, the SO, SR, and SLM exhibited increased GFAP expression (Fig. 10e, h, two-way ANOVA, interaction F(_3,24_) = 1.47, *p* = 0.247; layer F(_3,24_) = 17.38, *p* < 0.0001; alcohol F(_1,24_) = 44.65, *p* < 0.0001; Bonferroni post hoc test: SO *p* = 0.001; SR *p* = 0.048; SLM *p* = 0.0007). Of note, the SP in the CA3 and CA1 and the GCL in the DG did not show significant increases in GFAP expression.

On P45, there was a significant overall increase in GFAP expression in the DG (Fig. 10i, j, two-way ANOVA, interaction F_(2,18_) = 0.54, *p* = 0.591; layer F(_2,18_) = 6.92, *p* = 0.006; alcohol F(_1,18_) = 5.70, *p* = 0.028) and CA3 (Fig. 10i, k, two-way ANOVA, interaction F(_3,24_) = 0.11, *p* = 0.953; layer F(_3,24_) = 0.84, *p* = 0.487; alcohol F(_1,24_) = 11.35, *p* = 0.003) subregions, although post hoc analysis indicated that no individual layers exhibited a significant increase. Additionally, no increase was found in the CA1 subregion (Fig. 10i, l, two-way ANOVA, interaction F(_3,24_) = 0.26, p = 0.854; layer F(_3,24_) = 3.10, *p* = 0.046; alcohol F(_1,24_) = 1.73, *p* = 0.201).

## Discussion

This study is the first to perform a comprehensive investigation of neuroinflammation throughout multiple alcohol exposure and withdrawal episodes, during the third trimester-equivalent period of development. We report findings that complement earlier studies on the effects of PAE on the neuroimmune system. Specifically, we find vast differences in the hippocampus and cerebellar vermis with respect to cytokine production, neuronal loss, and glial cell activation. We report that signs of activation of both microglia and astrocytes occurred in parallel with neurodegeneration in the cerebellar vermis and with gait disturbances, while in the hippocampus, indications of astrocyte activation were evident in conjunction with spatial memory alterations. Intriguingly, at no measured point in the cerebellar vermis was there significant microglial presence within the Purkinje cell layer, where the majority of cell death transpired. Additionally, PAE increased cytokine expression in both brain regions, albeit to a lesser extent in the hippocampus. Interestingly, we find that, rather than building with subsequent exposures, cytokine production is transiently increased during withdrawal periods and a different response is elicited after one binge episode compared to three. The cytokines produced in this model were primarily pro-inflammatory, with only minor elevations in anti-inflammatory cytokines, and increases in either pathway were not long-lasting.

### Increased mRNA expression of pro-inflammatory cytokines occurs in the hippocampus and cerebellar vermis but only during periods of withdrawal

In the hippocampus and cerebellar vermis, we confirm that PAE stimulates production of pro-inflammatory cytokines. Surprisingly, cytokine production did not escalate as a function of exposure but rather spiked during periods of withdrawal (P4 and P6) and dropped off when BACs were at peak levels at the end of 4-h exposure periods (P3 and P5) (Figs 4a and 8a). Withdrawal from alcohol is associated with processes known to stimulate neuroinflammation, such as excitotoxicity [55], prevention of which has been shown to alleviate behavioral deficits in a PAE model [56]. Furthermore, withdrawal from binge exposure increases the number of microglia in adult rat brains [57]. As such, it is likely that withdrawal, rather than alcohol itself, directly contributes to cytokine production in the developing brain, although we cannot discount the possibility that this is in part explained by a delayed onset of cytokine production induced by alcohol exposure.

Additionally, it has been suggested that anti-inflammatory cytokines contribute to the alcohol-induced neuroimmune response in adults [10]. Therefore, we included anti-inflammatory cytokines in our analysis. While small increases were seen in TGFβ and IL10 in the cerebellar vermis and hippocampus, respectively, the majority of cytokine production was pro-inflammatory. However, anti-inflammatory cytokines could be responsible for the diminished pro-inflammatory response over the course of the paradigm as both TGFβ and IL10 can blunt the production of pro-inflammatory cytokines [58–60].

Our findings in the cerebellar vermis are consistent with previous observations that just 1 day of heavy PAE can increase levels of IL-1β in this region and that multiple days of exposure amplifies this effect [24]. Interestingly, while we also found TNFα expression to be elevated during the first withdrawal period, levels were not significantly above those in control animals after three withdrawal periods. This suggests that, in this case, the IL-1β and TNFα activation pathways are differentially affected. It is also possible that if anti-inflammatory cytokines are influencing pro-inflammatory cytokine expression as described above, they could elicit a greater effect on TNFα expression.

While the exact mechanism by which alcohol activates the neuroimmune system is not fully understood, particularly in the context of developmental exposure, studies in adults have shown that alcohol likely activates microglia through toll-like receptor 4 [61]. Moreover, alcohol exposure can increase secretion of the endogenous cytokine high mobility group box 1 (HMGB1) by neurons and other cells [62]. HMGB1 then activates toll-like receptor 4, expressed on microglia, and induces cytokine secretion in a positive feedback loop. It is possible that the mechanism for microglial activation by alcohol in the developing brain is similar.

### Regional differences between the hippocampus and cerebellar vermis may be a result of the window of exposure

Specific brain regions are susceptible to alcohol-induced damage, particularly neuronal loss, only during limited developmental windows. In the cerebellum, Purkinje cell loss is associated with exposure on P4-5 but not later [2, 63, 64], while the hippocampus is more resistant to cell loss during this period [65, 66]. Therefore, our exposure paradigm (P3–5) was expected to more heavily affect cells in the cerebellar vermis. In support of this, we saw no signs of neuronal loss in the hippocampus but massive Purkinje cell loss in the cerebellar vermis on P6. This loss was still evident on P45, although to a lesser extent. There was also a decrease in Purkinje cell number on P45 compared to P4 and P6, likely due to the period of programmed cell death that occurs in the early postnatal period (reviewed by [67]), as well as the expanding overall area of the cerebellum during development.

While there was dramatic Purkinje cell loss at the end of the three day exposure paradigm on P6, a single alcohol exposure on P3 had little effect on apoptotic signaling in the cerebellar vermis as assessed ~24 h later on P4. This is in contrast with previous findings showing a single day of exposure on P4 can increase caspase 3 signaling [68]. This disparity could be explained by the different methods of alcohol administration (injection versus inhalation). Additionally, in the previous study, alcohol was administered on P4, which is thought to be the day on which the cerebellum is most sensitive to insult by PAE [69, 70]. The P4 time point in our study was taken prior to alcohol exposure on P4 and therefore comprised animals exposed to alcohol only on P3. Finally, caspase 3 and TUNEL are both specific for markers of late apoptosis, so it is possible that the neurons in our study on P4 are in earlier stages of apoptosis, and future studies should address this possibility.

In the hippocampus, pro-inflammatory cytokine production was not as robust as in the cerebellar vermis. Of the cytokines measured, TNFα was the only one significantly increased on a specific day of the paradigm and only in the first withdrawal period. These observations challenge previous findings showing dramatically escalated levels of both pro- and anti-inflammatory cytokines in the hippocampus several weeks after exposure [25]. They also contrast with studies finding elevated levels of both TNFα and IL-1β in the hippocampus during withdrawal, 24 h after a P4-9 PAE paradigm [23]. However, in both cases, these discrepancies could be explained by differences in the method of administration (oral gavage versus vapor inhalation), rodent strain or type, the kind of sample collected (mRNA versus protein), and, in particular, the longer duration of the exposure paradigm. While our study specifically included a time window during which alcohol exposure more highly affects the cerebellum, the hippocampus has been shown to be more vulnerable to alcohol at a slightly later postnatal time point [71, 4, 51, 72], which overlaps with the exposure paradigms used in the studies described above.

Another important aspect of the timing of alcohol exposure, as it pertains to regional susceptibility, is the maturation rate of the neuroimmune system. During the early postnatal period, microglia mature faster from an amoeboid (activated) state to a branched (resting) morphological state in the hippocampus than in the cerebellum [73]. Indeed, we found that microglia in the hippocampus exhibited a more mature phenotype on P4 and P6 than those in the cerebellar vermis. Microglia that are amoeboid or in the early stages of transition could be primed to overreact to insult (reviewed by [74]), explaining the alcohol-induced increase in cytokine production in the cerebellar vermis, when compared to the hippocampus. Additionally, in agreement with previous studies [23, 26], by the end of our PAE paradigm, we observed a dramatic and obvious increase in the proportion of microglia in an amoeboid morphology in the cerebellar vermis, consistent with microglial activation. Conversely, changes in microglial morphology in the hippocampus were far more subtle. This is in contrast with observations in other models of PAE utilizing a longer exposure window that overlaps with hippocampal vulnerability [23]. Again, this is likely due to temporal differences in regional susceptibility and the maturation rate of microglia. These findings highlight the importance of considering the window of exposure when anticipating the effects of PAE on the neuroimmune system. Finally, it is possible that PAE-induced changes in morphology are related to the maturation of microglia, particularly within the hippocampus where the slight decrease in resting microglia on P6 could easily be explained by a delayed maturation of microglia following PAE, rather than activation. This possibility is less likely within the cerebellar vermis, where the proportion of microglia in an amoeboid morphology on P6 is increased compared to P4.

### PAE-induced increases in astrocytic GFAP expression occur with or without microglial morphological transitions: possible relationships to neurodegeneration

As stated above, neuronal loss was seen with this paradigm in the cerebellar vermis but not in the hippocampus. Interestingly, an increase in amoeboid microglia occurred only in the cerebellar vermis, while robust elevations in astrocytic GFAP expression was observed in both brain regions. While microglial morphological transitions and increased astrocytic GFAP expression do not always equate to activation, they provide considerable evidence of neuroinflammation and are highly associated with reactive glial cell functions [75–78]. Based on this, our findings support the idea that microglial activation is strongly associated with PAE-induced neuronal loss; however, whether microglial activation contributes to or is caused by it remains unclear. In support of the latter, there was little presence of microglia, amoeboid or otherwise, in or near the cell layer in which neurodegeneration occurred. However, it is possible that secreted factors, such as cytokines, could diffuse from a distance to directly affect Purkinje cell soma or damage the axons of these neurons, ultimately leading to neurodegeneration. Indeed, both TNFα and IL1β are capable of directly activating apoptotic pathways in neurons by binding to their respective receptors (reviewed by [79, 80]). Furthermore, increased cytokine mRNA levels did precede neuronal loss in the cerebellar vermis, although there were no signs of neuronal loss in the hippocampus, which also had significantly elevated levels of TNFα on P4. Aside from neuroinflammation, there exist many other ways in which neurons could be damaged following PAE, such as excitotoxicity, which has been shown to occur during alcohol withdrawal periods [55], as discussed above. Excitotoxicity can lead to severe oxidative stress, mitochondrial damage, and activation of apoptotic pathways (reviewed by [81]). Additionally, alcohol has been shown to directly induce endoplasmic reticulum stress in the postnatal brain (reviewed by [82]), and alcohol metabolism can generate harmful reactive oxygen species in the developing CNS (reviewed by [83]). Therefore, alcohol and/or alcohol withdrawal could be directly damaging neurons, independently of the neuroimmune system. Glial cell activation could then be secondary to neuronal distress, potentially through the early release of alarmin molecules such as HMGB1.

Reactive astrocytes were observed alongside neuronal loss and microglial morphological changes in the cerebellar vermis. However, unlike microglia, there was a robust increase in astrocytic GFAP expression in the PCL, where neurodegeneration occurred, although this increase was not markedly higher than in the other cell layers. Importantly, signs of astrocyte activation were also evident in the hippocampus, where there was no neuronal loss and little indication of microglial activation. In this brain region, the increase in astrocytic GFAP expression was lowest surrounding neuron somata. Taken together, these findings indicate that astrocytes react to PAE in a brain region-specific manner.

We observed strong signs of astrocyte activation following PAE, which demonstrates that astrocytes are very reactive to this environment and could indicate a potential role for astrocytes in the PAE-induced neuroinflammatory response. However, this study does not provide conclusive evidence that astrocytes or microglia influence PAE outcomes. Future studies should investigate whether the PAE withdrawal-associated increases in pro-inflammatory cytokine expression take place in astrocytes, microglia, and/or infiltrating peripheral immune cells.

Astrocyte activation varies heavily based on the type of insult and neighboring environmental cues (reviewed by [84]). As such, if glial cells are playing a role in PAE, it is likely that astrocytes in the hippocampus and cerebellar vermis have distinct functions. In some cases, astrocytes can be heavily anti-inflammatory, can restrict migration of local and peripheral inflammatory cells, and can secrete growth factors. Conversely, astrocytes can also have pro-inflammatory functions including secretion of IL-1β and TNFα (reviewed by [84]). Moreover, astrocytes have been shown to regulate microglial activation [29]. It is possible that astrocyte activation in the hippocampus prevents microglial activation and subsequent neuronal loss. Indeed, astrocytes have been shown to have many positive effects on neurons after alcohol exposure in cell culture and in vivo prenatal models [85–87]. For instance, as astrocytes are often strongly associated with increased neuronal survival [88, 89], they could also be directly preventing neuronal loss.

Finally, we cannot discount the possibility that there is some contribution by invading peripheral immune cells, such as macrophages. Astrocyte activation, as seen in this model, can be indicative of astrocytic scars, which can block migration of peripheral immune cells (reviewed by [84]). However, the blood brain barrier is not fully formed during the early postnatal period [90] making it easier for peripheral cells to infiltrate the brain. Additionally, the recent discovery of a novel lymphatic system, which allows the passage of peripheral immune cells into the CNS, has further implicated these cells in many CNS processes [91]. Finally, increased levels of pro-inflammatory cytokines, such as those seen in this model, can recruit peripheral immune cells (reviewed by [92]), and future studies should address this possibility.

### PAE results in deficits in both hippocampal and cerebellar-dependent behavior

PAE animals exhibited deficits in contextual fear conditioning, which is hippocampal dependent [93, 94]. Although we did not see signs of neuronal loss in the hippocampus, previous studies have identified several other mechanisms of PAE-induced damage in this brain region, including alterations in synaptic plasticity and transmission [95, 96], which could contribute to the observed deficit. Interestingly, both of these processes have been shown to be affected by TNFα [97–99] which we found to be up-regulated in the hippocampus of PAE rats. Therefore, neuroinflammation could be causing hippocampal-dependent deficits through more subtle alterations, rather than through cell loss. On the other hand, while contextual fear conditioning is hippocampal-dependent, performance on this task may also require other brain regions such as the amygdala and frontal cortex (reviewed by [100]). Therefore, PAE could induce alterations in fear conditioning through a non-hippocampal mechanism.

Additionally, PAE animals had significant alterations in gait. While gait has been shown to be highly dependent on the cerebellum and particularly on Purkinje cells [49, 48], it is also affected in other types of neurodegenerative diseases that have more widespread effects in the brain [50, 101, 102]. Therefore, while it is likely that PAE-induced Purkinje cell loss heavily impacts gait in these animals, this paradigm may also cause damage to other structures, such as descending spinal pathways or motor cortex, which could further contribute to alterations in gait. Whether or not neuroinflammation is directly contributing to these deficits remains unclear as the nature of the relationship between neuroimmune activation and neuronal damage/loss requires further research.

### Overall conclusions

In summary, this study provides information that fills the following gaps in knowledge within this field: (1) that PAE induces neurodegeneration, neurobehavioral deficits, and neuroimmune activation in a region- and time-specific manner rather than globally; (2) that the cytokine response to PAE is primarily pro-inflammatory and transient, mainly occurring during alcohol withdrawal periods; and (3) that not only microglia but also astrocytes become activated following developmental alcohol exposure. Future work should investigate the mechanisms underlying these region- and time-specific effects of PAE, as well as the potential utility of anti-inflammatory agents in the treatment of FASDs.

## Additional files

Additional file 1:
**Table providing a description of the parameters used in gait assessment.** (TIFF 753 kb) Additional file 2:
**Table providing additional measurements of gait in PAE and control animals.** (TIFF 636 kb) Additional file 3:
**Postnatal alcohol exposure (PAE) does not affect granule cell layer thickness in the cerebellar vermis.** Effect of PAE on external granule layer (EGL) and internal granule layer (IGL) thickness in the cerebellar vermis during the first withdrawal period on P4 (a–b), the third withdrawal period on P6 (c–d) (see Fig. 1a). Additionally, the granule cell layer is measured on P45 (e). Animals were treated as described in Fig. 1. To investigate regional difference, lobules I–X of the cerebellar vermis were grouped into three lobule regions and quantified separately. For sample images, see Fig. 3. *n* = 4 animals from 4 litters. (TIFF 279kb) Additional file 4:
**Postnatal alcohol exposure (PAE) does not increase activated caspase in the cerebellar vermis.** Effect of PAE on activated caspase 3 expression in the cerebellar vermis during the first withdrawal period on P4 (see Fig. 1a). Animals were treated as described in Fig. 1. Representative images of the cerebellar vermis are stained for cleaved caspase 3 (red) to label late apoptotic neurons and 4′,6-diamidino-2-phenylindole (DAPI, blue) to label cell nuclei. To investigate regional difference, lobules I–X of the cerebellar vermis were grouped into three lobule regions and staining intensity was quantified within the external granule layer (EGL), Purkinje cell layer (PCL), and internal granule layer (IGL). *n* = 4 animals from 4 litters. Scale bar = 40 μm. (TIFF 2,029 kb) Additional file 5:
**Postnatal alcohol exposure (PAE) does not increase TUNEL staining in the cerebellar vermis.** Effect of PAE on TUNEL staining in the cerebellar vermis during the first withdrawal period on P4. Animals were treated as described in Fig. 1. Representative images of the cerebellar vermis were stained with a TUNEL assay (red) to label apoptotic neurons and 4′,6-diamidino-2-phenylindole (DAPI, blue) to label cell nuclei. To investigate regional difference, lobules I–X of the cerebellar vermis were grouped into three lobule regions and staining intensity was quantified within the external granule layer (EGL), Purkinje cell layer (PCL), and internal granule layer (IGL). (b) *n* = 4 animals from 4 litters. Scale bar = 40 μm. (TIFF 2,323 kb)

## Abbreviations

BAC: blood alcohol concentration
CT: threshold cycle value
DAPI: 4′, 6-diamidino-2-phenylindole
DG: dentate gyrus
EGL: external granule layer
FASD: fetal alcohol spectrum disorder
GCL: granule cell layer
GFAP: glial fibrillary acidic protein
HMGB1: high mobility group box 1
HPRT1: hypoxanthine phosphoribosyltransferase 1
IBA-1: ionized calcium-binding adapter molecule 1
IGL: internal granule layer
IL10: interleukin-10
IL1β: interleukin-1β
LF: left forepaw
LH: left hind paw
ML: molecular layer
P: postnatal day
PAE: postnatal alcohol exposure
PBS: phosphate buffered saline
PCL: Purkinje cell layer
RF: right forepaw
RH: right hind paw
SL: stratum lucidum
SLM: stratum lacunosum moleculare
SO: stratum oriens
SP: stratum pyramidale
SR: stratum radiatum
T1: transitional 1
T2: transitional 2
TGFβ: transforming growth factor β
TNFα: tumor necrosis factor α
TUNEL: terminal deoxynucleotidyl transferase dUTP nick-end labeling
