# Supplementary figures and images for: Exposure of neonatal rats to alcohol has differential effects on neuroinflammation and neuronal survival in the cerebellum and hippocampus
Source: J Neuroinflammation. 2015 Sep 4;12:160. doi: 10.1186/s12974-015-0382-9 (PMC4558631; doi:10.1186/s12974-015-0382-9)

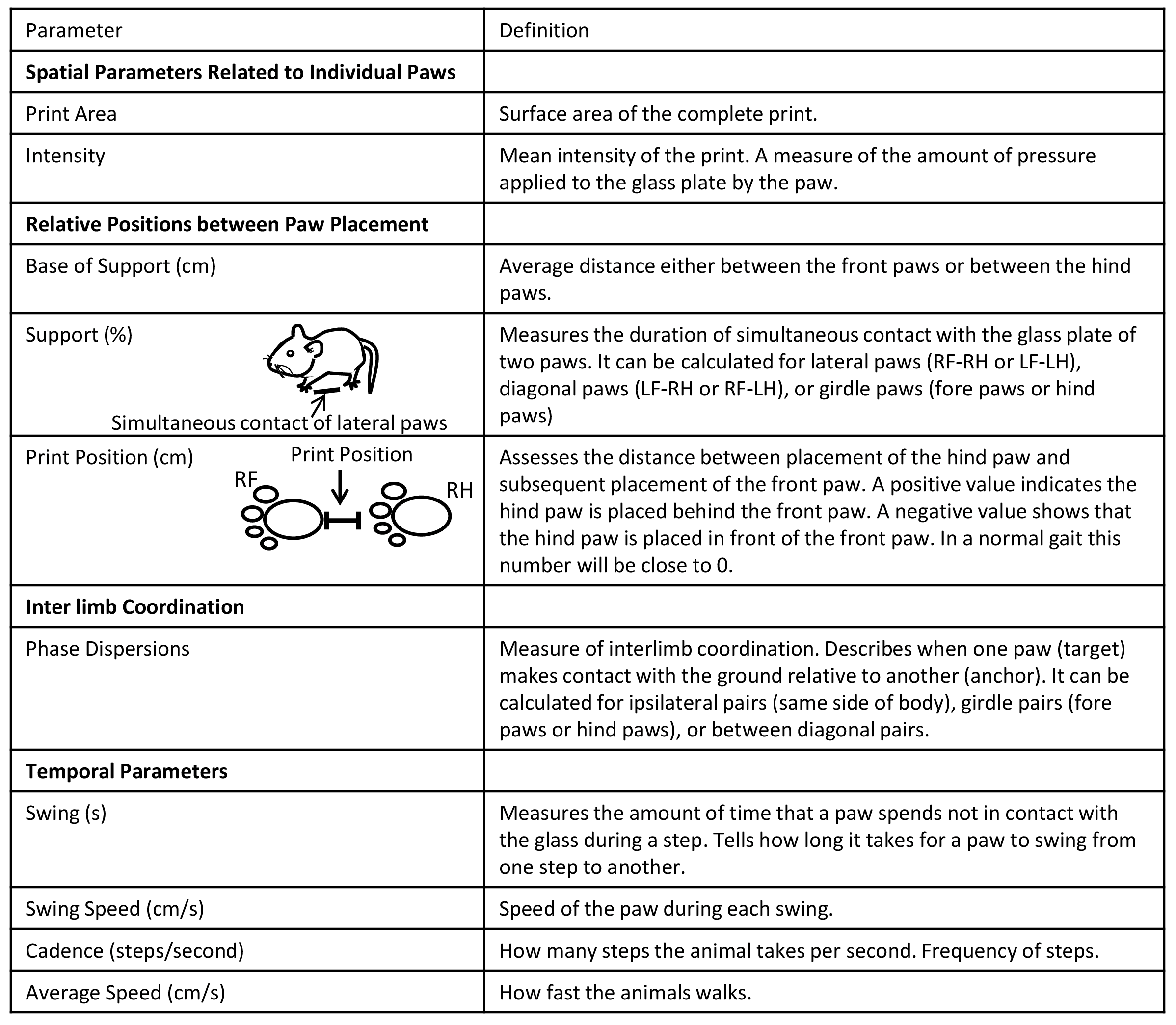

Supplement: Additional file 1: — Table providing a description of the parameters used in gait assessment. (TIFF 753 kb) [file 12974_2015_382_MOESM1_ESM.tiff]

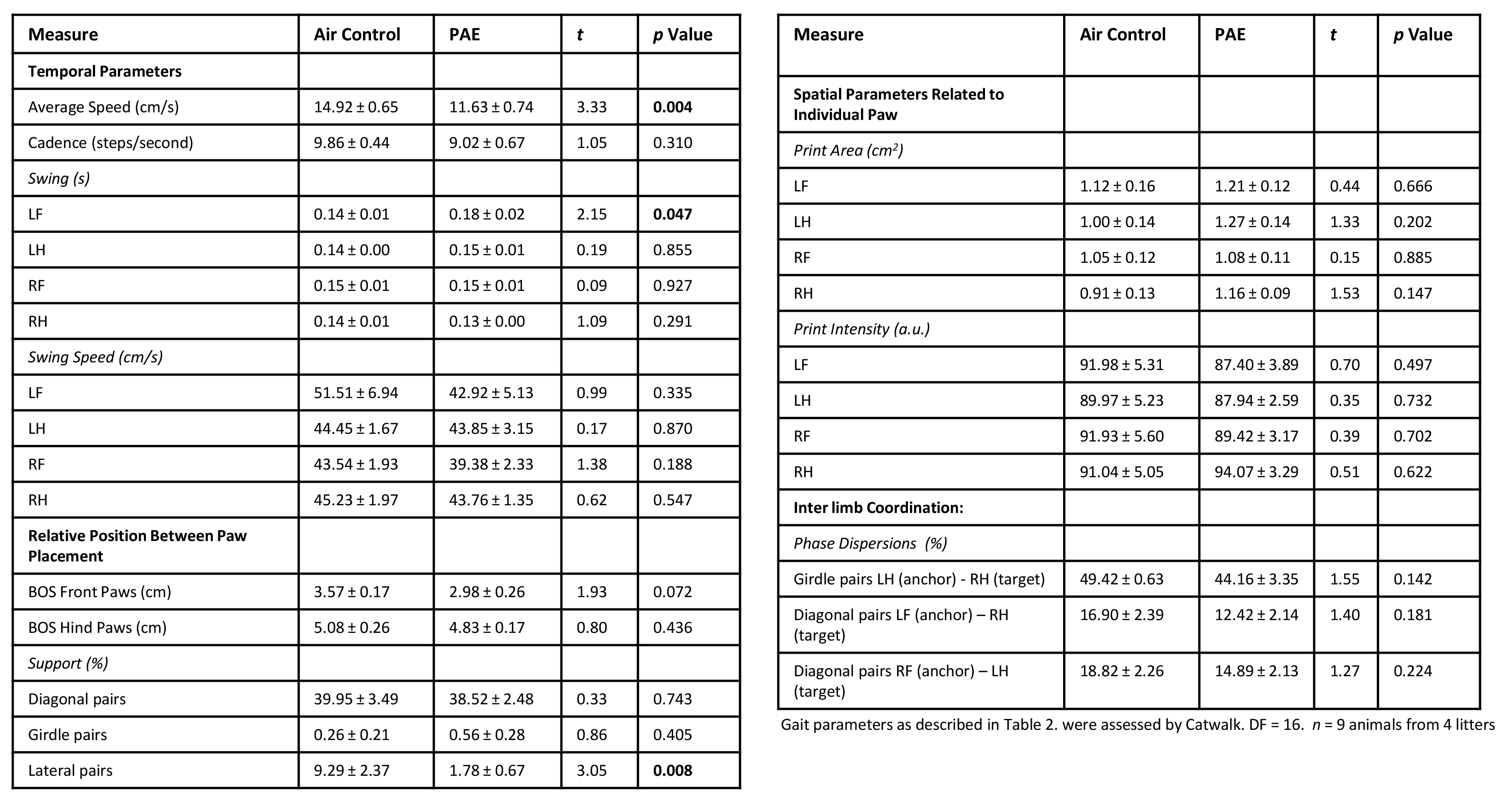

Supplement: Additional file 2: — Table providing additional measurements of gait in PAE and control animals. (TIFF 636 kb) [file 12974_2015_382_MOESM2_ESM.tiff]

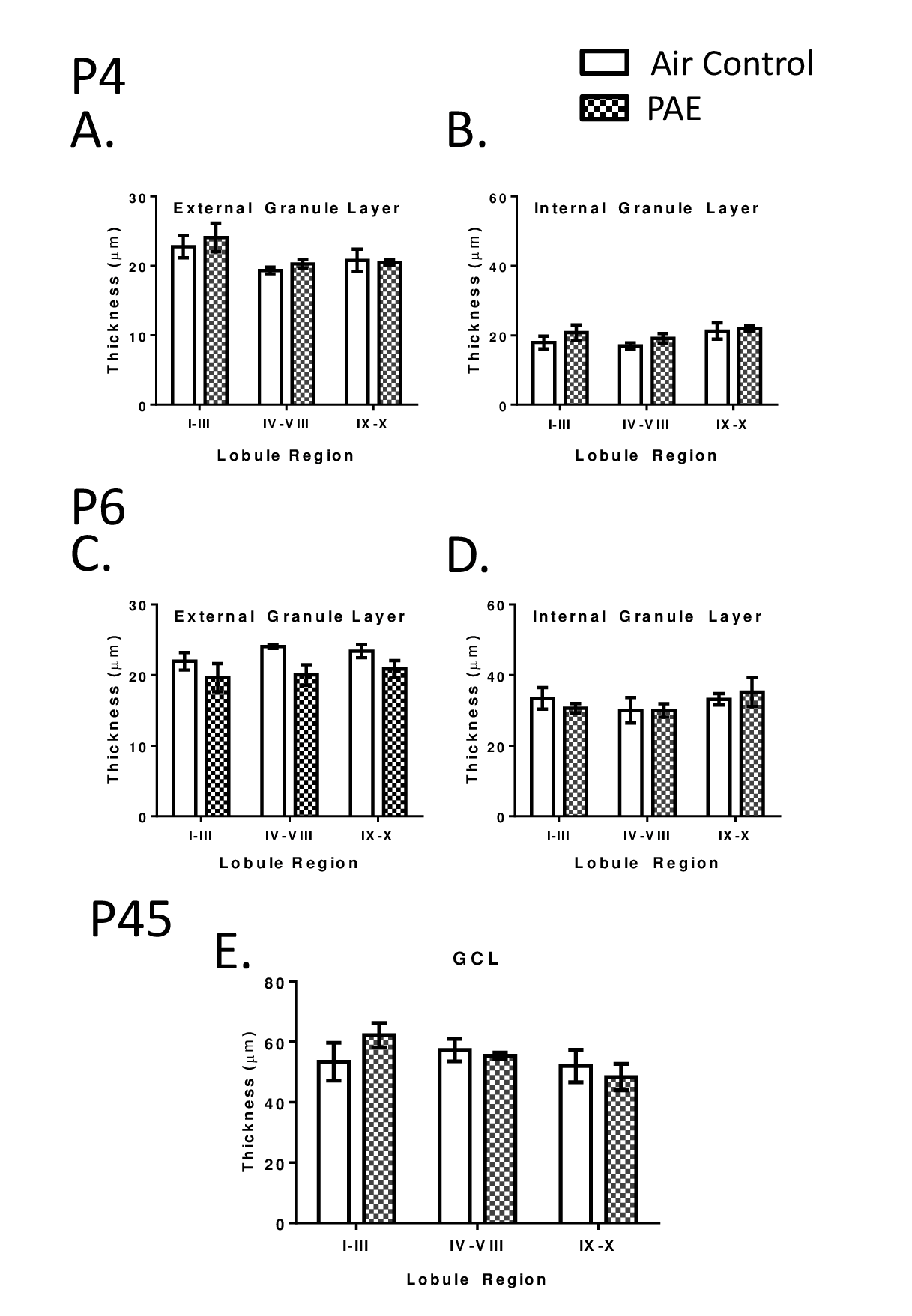

Supplement: Additional file 3: — Postnatal alcohol exposure (PAE) does not affect granule cell layer thickness in the cerebellar vermis. Effect of PAE on external granule layer (EGL) and internal granule layer (IGL) thickness in the cerebellar vermis during the first withdrawal period on P4 (a–b), the third withdrawal period on P6 (c–d) (see Fig. 1a). Additionally, the granule cell layer is measured on P45 (e). Animals were treated as described in Fig. 1. To investigate regional difference, lobules I–X of the cerebellar vermis were grouped into three lobule regions and quantified separately. For sample images, see Fig. 3. n = 4 animals from 4 litters. (TIFF 279kb) [file 12974_2015_382_MOESM3_ESM.tiff]

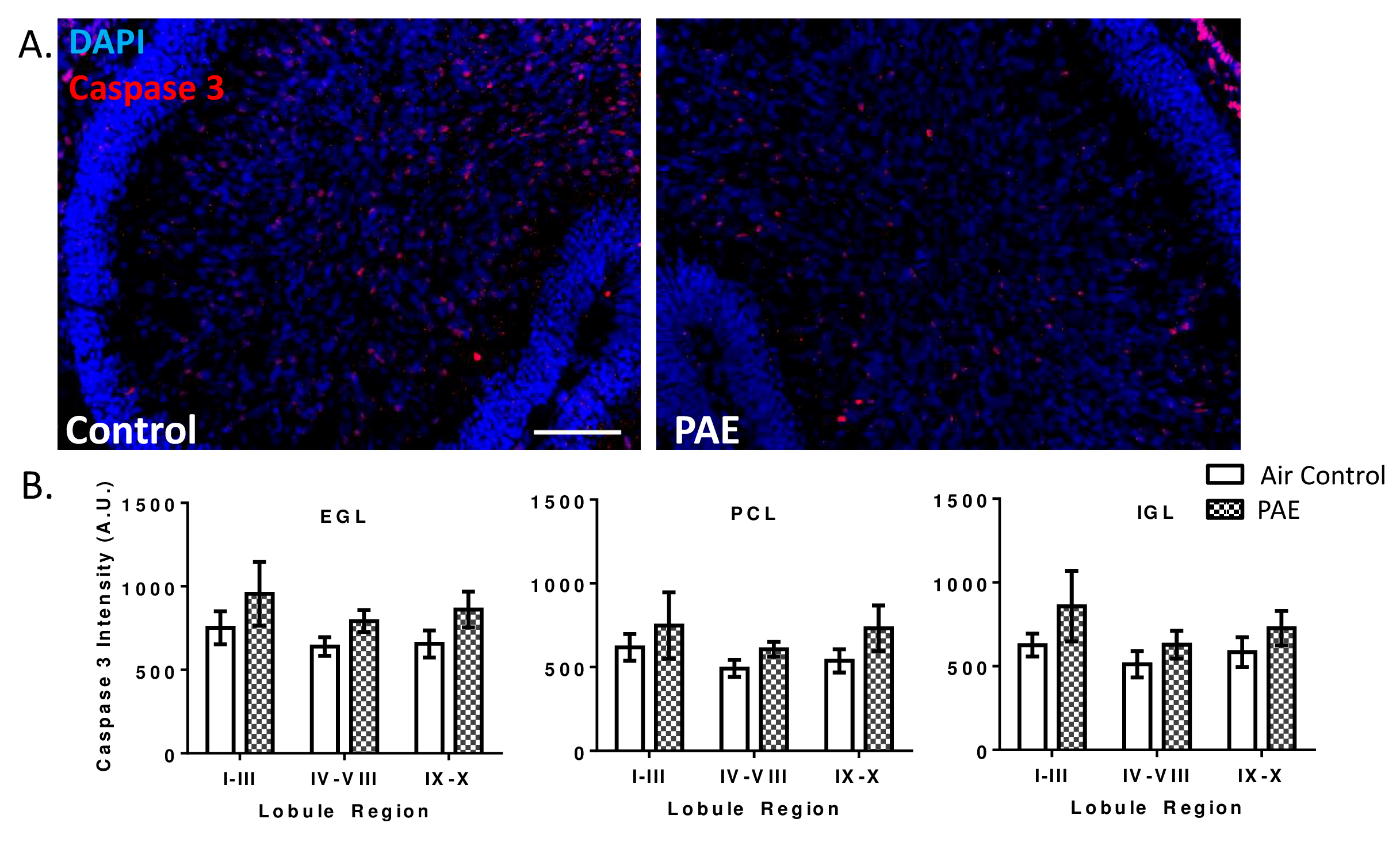

Supplement: Additional file 4: — Postnatal alcohol exposure (PAE) does not increase activated caspase in the cerebellar vermis. Effect of PAE on activated caspase 3 expression in the cerebellar vermis during the first withdrawal period on P4 (see Fig. 1a). Animals were treated as described in Fig. 1. Representative images of the cerebellar vermis are stained for cleaved caspase 3 (red) to label late apoptotic neurons and 4′,6-diamidino-2-phenylindole (DAPI, blue) to label cell nuclei. To investigate regional difference, lobules I–X of the cerebellar vermis were grouped into three lobule regions and staining intensity was quantified within the external granule layer (EGL), Purkinje cell layer (PCL), and internal granule layer (IGL). n = 4 animals from 4 litters. Scale bar = 40 μm. (TIFF 2,029 kb) [file 12974_2015_382_MOESM4_ESM.tiff]

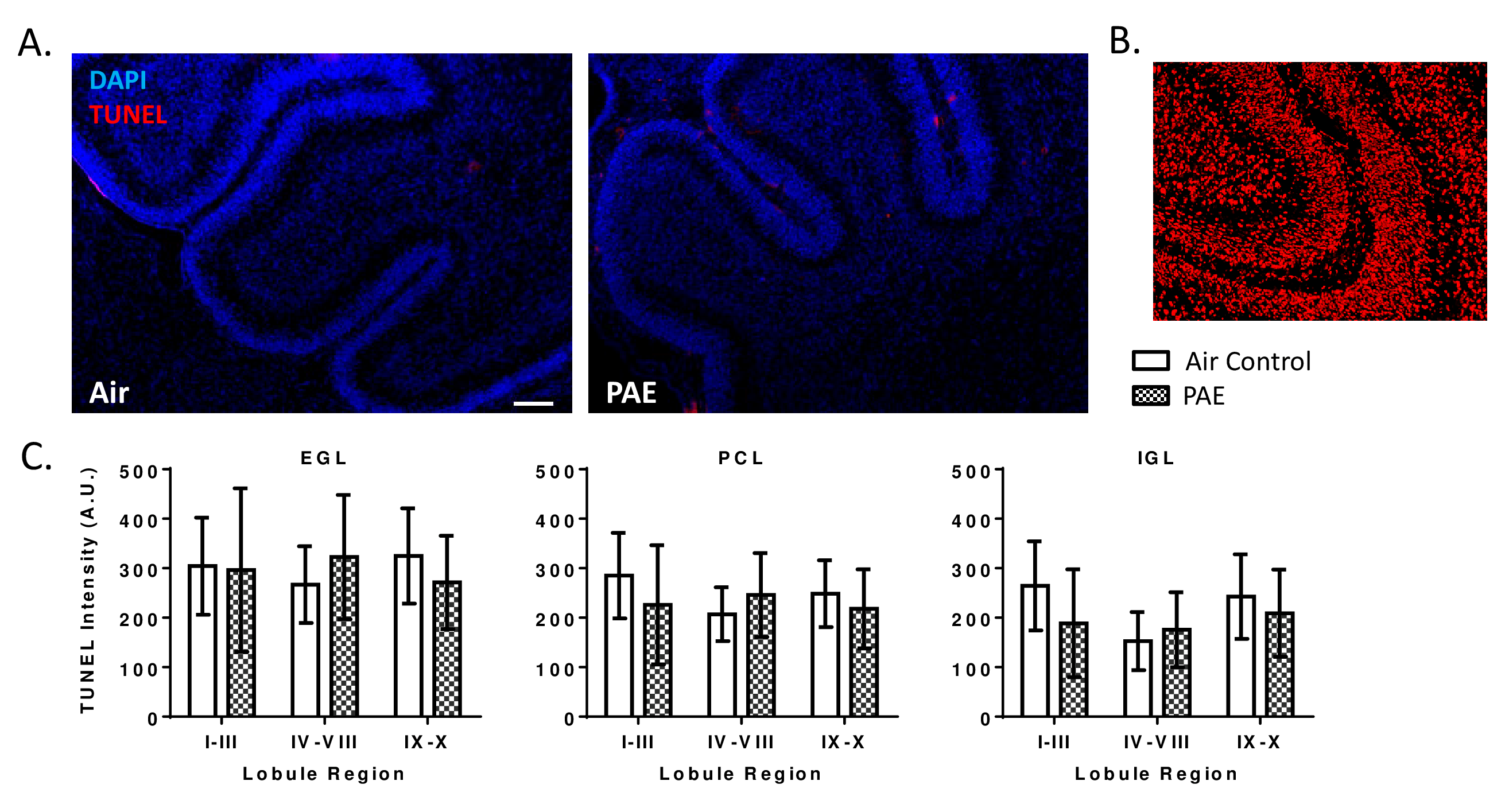

Supplement: Additional file 5: — Postnatal alcohol exposure (PAE) does not increase TUNEL staining in the cerebellar vermis. Effect of PAE on TUNEL staining in the cerebellar vermis during the first withdrawal period on P4. Animals were treated as described in Fig. 1. Representative images of the cerebellar vermis were stained with a TUNEL assay (red) to label apoptotic neurons and 4′,6-diamidino-2-phenylindole (DAPI, blue) to label cell nuclei. To investigate regional difference, lobules I–X of the cerebellar vermis were grouped into three lobule regions and staining intensity was quantified within the external granule layer (EGL), Purkinje cell layer (PCL), and internal granule layer (IGL). (b) n = 4 animals from 4 litters. Scale bar = 40 μm. (TIFF 2,323 kb) [file 12974_2015_382_MOESM5_ESM.tiff]
